# Proportion-based normalizations outperform compositional data transformations in machine learning applications

**DOI:** 10.1186/s40168-023-01747-z

**Published:** 2024-03-05

**Authors:** Aaron Yerke, Daisy Fry Brumit, Anthony A. Fodor

**Affiliations:** 1 Department of Bioinformatics and Genomics, Bioinformatics Building, UNC Charlotte, The University of North Carolina, Charlotte 9331 Robert D. Snyder Rd, Charlotte, USA; 2 Food Components and Health Laboratory, USDA, ARS, Beltsville Human Nutrition Research Center, Beltsville, USA

**Keywords:** Metagenomics, Statistical data interpretation, Compositional data, Machine learning, Random forest, High-throughput nucleotide sequencing, Transformation, Normalization, PhILR

## Abstract

**Background:**

Normalization, as a pre-processing step, can significantly affect the resolution of machine learning analysis for microbiome studies. There are countless options for normalization scheme selection. In this study, we examined compositionally aware algorithms including the additive log ratio (alr), the centered log ratio (clr), and a recent evolution of the isometric log ratio (ilr) in the form of balance trees made with the PhILR R package. We also looked at compositionally naïve transformations such as raw counts tables and several transformations that are based on relative abundance, such as proportions, the Hellinger transformation, and a transformation based on the logarithm of proportions (which we call “lognorm”).

**Results:**

In our evaluation, we used 65 metadata variables culled from four publicly available datasets at the amplicon sequence variant (ASV) level with a random forest machine learning algorithm. We found that different common pre-processing steps in the creation of the balance trees made very little difference in overall performance. Overall, we found that the compositionally aware data transformations such as alr, clr, and ilr (PhILR) performed generally slightly worse or only as well as compositionally naïve transformations. However, relative abundance-based transformations outperformed most other transformations by a small but reliably statistically significant margin.

**Conclusions:**

Our results suggest that minimizing the complexity of transformations while correcting for read depth may be a generally preferable strategy in preparing data for machine learning compared to more sophisticated, but more complex, transformations that attempt to better correct for compositionality.

Video Abstract

**Supplementary Information:**

The online version contains supplementary material available at 10.1186/s40168-023-01747-z.

## Background

Machine learning application to metagenomic data has been used to successfully predict a variety of phenomena from states of human health, such as cancer [1] and gastrointestinal diseases [2], to environmental and ecological conditions such as soil health [3] and carbon dynamics [4]. Improving the accuracy of these predictions would therefore provide benefits to multiple fields of research. The goal of data transformations is to improve accuracy by reducing artifacts and noise in the data. Two notorious sources for artifacts are compositionality and the arbitrary nature of sequence read depth [5, 6].

Compositionality is a well-known problem in sequencing experiments [5]. Compositional data are any data that are considered to be parts of a whole [7]. As such, independence between values cannot be assumed because if one part increases in value, another must decrease due to the constraint of all parts summing to a fixed constant. In the case of current DNA sequencing technologies such as Illumina Miseq, the fixed amount that sequences sum to is the read depth, which varies from sample to sample [5]. This is a problem for inference statistics because they assume independence and vectors of features that sum to a constant are, by definition, not independent.

Compositionally aware transformations attempt to address this problem such that the transformed data can be used with inference statistics. Three compositionally aware transformations that we explore in this article are the additive log ratio (alr), the centered log ratio (clr), and the isometric log ratio (ilr). Each of these transformations is based on the principle that the product of a log ratio is transformed to real space, breaking the sample space of the compositional data out of a constrained hyperplane and making it appear independent. The alr accomplishes this by selecting a single feature and using it as the denominator in the ratio [7]. The output of this transformation is n-1 in size where the number of features is n, as the feature used in the denominator is never used in the numerator. In the case of sequencing data, the features are the taxa or amplicon sequence variants (ASVs). The clr is very similar to the alr but uses the geometric mean of all the features as the denominator in the log ratio [7]. The ilr uses ratios of arbitrarily selected features in its log ratio; thus, input and output cannot be assumed to be directly related to single features [8].

The number of possible ilr implementations increases factorially with the features in the dataset [9]. Thus, for highly dimensional datasets, such as those used in metagenomics, it becomes essentially impossible to calculate every possible ilr variation. A solution to the very large number of ilr transformations was independently proposed by two groups who utilized phylogenetic data to guide the ilr transformations [10, 11]. PhILR is a popular R package for creating such ilr transformations, which are sometimes referred to as balance trees. PhILR additionally offers two weighting schemes; however, the benefits of these weighting schemes to machine learning remain unclear.

Numerous previous studies have used compositionally aware transformations to transform data prior to analysis with machine learning algorithms (MLAs) [12–16]. However, it is unclear if these transformations offer benefits to machine learning and, if so, which transformation or weighting schemes should be utilized. In this article, we examine 4 publicly available 16S rRNA sequencing datasets containing a total of 1798 samples and 65 metadata features to compare popular transformations that are compositionally aware to transformations that are compositionally naïve (i.e., treat sequence data as raw counts or unconstrained proportions). These transformations include raw counts tables, rarefaction, proportions, the Hellinger transformation, and a procedure we call “lognorm,” which is based on the log-proportion scaled to the average sequencing depth and an added pseudo-count of 1. The latter three are all based on creating a relative abundance using read depth as a reference.

We show that no weighting scheme or combination of weighting schemes is more consistently effective than another for balance trees. We find, somewhat surprisingly, that using read depth-based relative abundances reliably produces small but statistically significant improvements over compositional data transformations. Also surprisingly, not transforming the data at all (i.e., utilizing raw count tables not correcting for read depth) also consistently outperformed compositionally aware transformations. We conclude that, while the reasoning behind compositional transformation is compelling, more straightforward transformations appear to often be more effective for machine learning classification of many metadata variables.

## Materials and methods

For this project, we chose four publicly available 16S rRNA gene sequencing datasets for our analysis with sample sizes ranging from 233 to 700 (Table 1). From each dataset, we dropped sample metadata features that were sparse (< 1/4 total samples) yielding a total of 65 metadata categories across all 4 datasets (Table 1) (additional details at Project additional information in supplemental files).

**Table 1:** Datasets used in this study

Name, reference, and accession number	Number of samples	Metadata categories
Vangay [17] PRJEB28687	634	Recruitment.Location, Researcher, Sub.Study, Birth.Year, Age, Highest.Education, Ethnicity, Religion, Birth.Location, Type.Birth.Location, Arrival.in.US, Years.in.US, Location.before.US, Type.location.before.US, Years.lived.in.Location.before.US, Tobacco.Use, Alcohol.Use, HeightWeight, Waist, BMI, BMI.Class, Breastfed, Age.at.Arrival, Sample.Group, Waist.Height.Ratio
Jones [18] PRJNA397450	233	Age, BMI, Genotype, sex, Treatment, Visit, type
Zeller [13] PRJNA397450	226	Age, host_subject_id, geographic_location_(country_and/or_sea region), Collection_date, AJCC_Stage, localization, tissue_type
Noguera-Julian [19] PRJNA307231	700	Host_Age, ETHNICITY, geo_loc_name_country, HIV_RiskGroup, HIV_serostatus, host_other_gender, host_sex, HIV_Profile, PCR_human_papilloma_virus, host_allergy, host_deposition_frequency_per_day, host_abdominal_transit_alterations, host_Residency_Area, HCV_coinfection, Anal_cytology, host_sexual_orientation, Syphilis_serology, HBV_coinfection, PCR_Neisseria_gonorrhoeae, PCR_Chlamydia_trachomatis, HIV_viral_load, CD4 + _Tcell_counts, leukocytes, stool_consistency, lymphocytes, host_body_mass_index

### Sequence processing

For 16S sequencing, we only used the forward reads, as the reverse reads tend to have a higher error rate [20]. We filtered, trimmed, removed bimeras, and assigned taxonomy to the 16S sequences with version 1.0.3 of the R package DADA2. The resulting ASVs were aligned using version 2.0.2 of the R package DECIPHER.

### Prevalence filtering

For the prevalence filtered datasets, we removed ASVs that were found in only 10% of the samples or less before downstream transformations.

### Tree building for PhILR

**Fig. 1:**
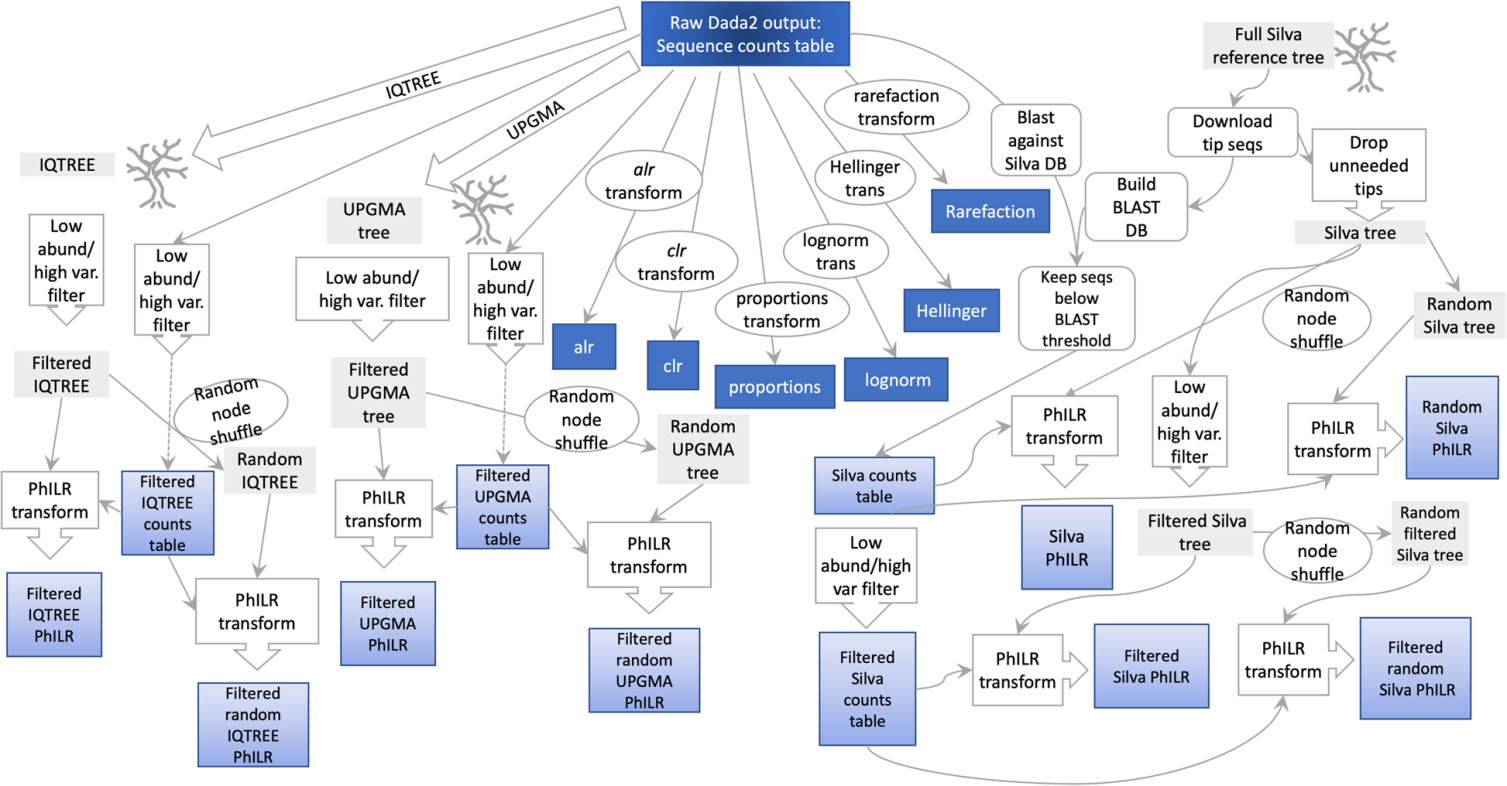
Schematic representation of workflow for the creation of data transformations from the raw Dada2 counts tables. This schematic starts with the raw Dada2 counts tables and each step in the workflow (grey arrows with white bubbles) lead to trees (grey blocks) or final datasets (blue boxes) as described in the methods section. Datasets with random node shuffles were recreated at least 3 times

The purpose of our study was to compare compositionally naïve and aware transformations. There were many steps taken to create all the transformations used in this project. We have provided a sketch of how each transformation was generated from raw counts tables (Fig. 1) and prevalence filtered counts tables (Supplementary B).

Balance trees made from sequencing data are an implementation of the ilr that is guided by bifurcating phylogenetic trees. The inputs for a balance tree are therefore a counts table and a phylogenetic tree. To determine how the choice of phylogenetic tree impacts the ability of the transformed data to train a random forest algorithm, we chose two de novo trees and one curated reference tree, the SILVA’s Living Tree Project (LTP) [21]. To use SILVA’s LTP reference tree, for each dataset in our study, we removed the taxa that were not present in that dataset from the SILVA LTP tree. For our de novo trees, we utilized both a simple “naïve” method Unweighted Pair Group Method with Arithmetic Mean clustering (UPGMA) that ignores variation in biological clocks and a more computationally intensive, sophisticated method -IQTREE. UPGMA is a method that clusters the sequences based on distance matrices [22]. Hierarchical clustering is often considered to be an overly simple approach, but we felt that it would be useful as a de novo control. IQTREE by contrast infers trees by maximum likelihood. A disadvantage of de novo methods, compared to the LTP tree, is that it will use all of the sequences available to it and will force branches between nodes based on limited data. Therefore, it will contain more nodes than the trees we made through subtractive process that we used for the Silva LTP trees. For every dataset, the SILVA trees were an order of magnitude smaller than the UPGMA and IQTEE trees (Table 2). Images of unfiltered SILVA LTP tree, the high variance/low abundance filtered SILVA LTP tree, the UPGMA, and IQTREE can be found in Supplementary A in the supplemental files.

**Table 2:** Tree descriptions

	Tree name	Num. nodes	Num. tips	Ave. branch length	Variance branch length	Ultrametric (root-tip distance equal for all tips)
Jones	SILVA	1132	1133	0.0311	0.0012	FALSE
	Filtered_SILVA	75	76	0.0556	0.0032	FALSE
	Filtered_UPGMA	227	228	0.0307	0.0015	TRUE
	UPGMA	28,025	28,026	0.0384	0.0104	TRUE
	IQTREE	28,024	28,026	0.1205	0.1798	FALSE
	Filtered_IQTREE	227	228	0.0840	0.0191	FALSE
Vangay	SILVA	906	907	0.0344	0.0012	FALSE
	Filtered_SILVA	35	36	0.1038	0.0130	FALSE
	Filtered_UPGMA	70	71	0.1300	0.0642	FALSE
	UPGMA	6821	6823	0.0521	0.3193	FALSE
	IQTREE	6821	6823	0.0202	0.0299	FALSE
	Filtered_IQTREE	70	71	0.0813	0.0144	FALSE
Zeller	SILVA	1490	1491	0.0309	0.0009	FALSE
	Filtered_SILVA	121	122	0.0606	0.0044	FALSE
	Filtered_UPGMA	207	208	0.0332	0.0012	TRUE
	UPGMA	11,077	11,078	0.0154	0.0007	TRUE
	IQTREE	11,076	11,078	0.0298	0.0409	FALSE
	Filtered_IQTREE	207	208	0.0792	0.0136	FALSE
Noguera-Julian	SILVA	1233	1234	0.0330	0.0011	FALSE
	Filtered_SILVA	52	53	0.0840	0.0089	FALSE
	Filtered_UPGMA	122	123	0.0273	0.0025	TRUE
	UPGMA	20,365	20,366	0.0509	0.0180	TRUE
	IQTREE	20,364	20,366	0.1204	0.1501	FALSE
	Filtered_IQTREE	122	123	0.0668	0.0295	FALSE

For each phylogenetic tree that we used for PhILR, we made 3 random shuffles of the nodes and included them as controls. The shuffled trees have the same number of nodes and tips, but their nodes do not match to the same tips as the true trees and their branch-lengths will be incorrect. This tests how important this information is to weighting schemes that use it.

#### UPGMA tree

To build the UPGMA trees de novo from the sequencing data, we used version 2.9.0 of the phangorn R package [23].

#### IQTREE

The alignment file from the DADA2 sequence processing was processed through the IQ-TREE version 2.1.2 for Linux 64-bit. We allowed the modelFinder to run for 48 h on the Jones dataset using 1 core and then selected the highest scoring model for subsequent runs. The highest scoring model was “GTR + F + R5,” which is a combination of a general time reversible model with unequal rates and unequal base, empirical amino acid frequencies, and the R5 free-rate model.

The resulting customized reference trees and the de novo trees were then available for building phyloseq objects using version 1.16.2 of the phyloseq R package. A phyloseq object consists of a single object that holds sequencing data, sequence metadata, a taxonomy, and a tree. The phyloseq objects were later used for the *philr* function from version 1.24.0 of the PhILR R package.

#### SILVA Living Tree Project

The reference tree comes from SILVA’s Living Tree Project, 16S rRNA-based LTP release 132 [21]. The reference tree lists the GenBank locus at each tip, so we used this information to download the sequences from GenBank using the ape package (Fig. 1). We then built a blast database out of the sequences and blasted the sequences from our study datasets using custom BASH scripts. If the resulting matches had e-value greater or equal to ${10}^{-10}$, we culled them from the tips of the reference tree using custom R scripts to get a customized reference tree (LTP tree). Though the exact value of ${10}^{-10}$ was chosen arbitrarily, we believe that it is an appropriate conservative threshold. We hypothesized that though the PhILR transformations made from SILVA LTP trees were smaller, they would perform better due to a filtering effect.

#### Variance and low abundance filtration and the PhILR transform

Corresponding pairs of trees and counts tables were low abundance filtered using the following criteria as mentioned in PhILR’s vignette:

$${\text{sum}}\left(x>3\right)>\left({0.2}^{*}{\text{length}}\left(x\right)\right)$$

**Fig. 2:**
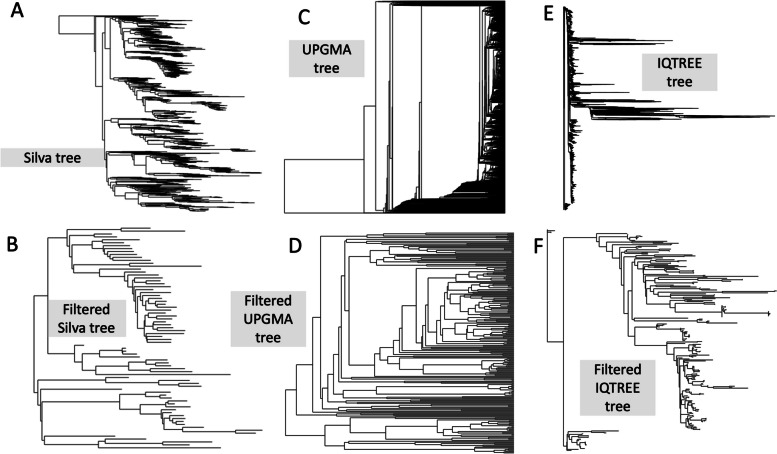
The low abundance/high variance filtered trees are less cluttered than the unfiltered originals in the Jones dataset. The unfiltered SILVA LTP reference tree (**A**) shows more taxa than the filtered version (**B**). The UPGMA (**C**) and IQTREE (**E**) algorithms are both greedy and thus the trees are too dense and were only used after low abundance/high variance filtering (**D** and **F**, respectively)

And then high variance samples meeting the following criteria were filtered:

$$sd\left(x\right)/{\text{mean}}\left(x\right)>3.0$$

where *x* is the sequence in the counts table, *sd* is standard deviation, and the length is the number samples—the sequences were also dropped from the tips of thecorresponding tree. Without this, the UPGMA and IQTREE trees were too large for PhILR; thus, we only used the filtered version of each of these trees in our experiments (Fig. 2). The sample counts were then given a pseudo count of 1 to eliminate the zeros that would impede PhILR. Finally, the phyloseq objects were processed through PhILR to create the ILR transform of our sequencing data tables.

#### Randomly shuffled trees

The phylogenetic trees consist of nodes and tips. The tips are the unique sequences of the DADA2 output, and their counts are provided by the counts tables. The nodes of the tree are a hierarchical cluster showing the ancestry of the sequences. All of the tips connected to that node share a common ancestor. The nodes are used by PhILR to group the ratios when creating balances. Thus, as a further control in this experiment to test how the quality of the phylogenetic tree affects PhILR, we created 3 randomly generated trees that have the same number of nodes and tips as the “true” trees. In these randomly generated trees, nodes on trees were connected to other nodes or tips irrespective of true phylogenetic distance or ancestry. This meant that the ratios used in the PhILR transform were no longer guided by an accurate phylogeny. The random trees are meant to examine how the phylogenetic trees affect the resolution of the PhILR transformed data.

The random trees were generated using the *rtree* function from version 5.6–2 of the ape R package [24].

#### PhILR weighting schemes

PhILR offers two weighting schemes—branch length weighting with four options and taxon weighting with six options. This gives a total of 24 combinations. The taxon weighting options are no weight, the geometric mean, the Aitchison norm, the Euclidean norm, and the geometric mean multiplied by either the Aitchison norm or the Euclidean norm. The authors of PhILR prefer the geometric mean multiplied by the Euclidean norm, as this performed well in their preliminary benchmarks [10].

The second weighting scheme weights the branches of the tree. Each balance can either be weighted by the sum of its children’s branch lengths, the square root of the sum of the children’s branch lengths, or the sum of children’s branch lengths plus the mean descendants each child’s mean distance to its descendent tips. These weights enable the PhILR transform to differentiate itself from a pure ilr transform [10].

### Non-tree CODA transformations

The *alr* and *clr* transformations of the ASV tables were done using the alr and clr functions, respectively, of version 2.0–6 of the R package called “compositions” [25]. For the denominator of that *alr*, the taxa that was present in the most samples, was selected. To prevent zeros in the denominator for both of these transformations, counts tables were augmented with a pseudo count of 1.

### Read depth-based transformations

Relative abundance or simple proportion is the simplest read depth-based transformation, which was implemented with a custom R script as per the formula:

$$\frac{RC}{n}$$

We implemented square root of relative abundance (Hellinger transformation) as per the following formula:

$$\sqrt{\frac{{\text{RC}}}{{\text{n}}}}$$

The lognorm transformation was implemented as a custom R script as per the formula:

$${{\text{log}}}_{10}\left(\frac{RC}{n}\times \frac{\sum x}{N}+PC\right)$$

where RC = raw counts in a cell, *n* = number of sequences in a sample, Σ*x* = total number of counts in the table, *N* = total number of samples, PC = pseudo-count, for this project was equal to 1. The $\frac{\sum x}{N}$ term in the lognorm equation is a constant that is the same for each element in the table. It serves to normalize dispersion between datasets. The pseudo count allows for the application of the logarithm to the sparse data.

### Rarefaction

Rarefaction was performed using the *rrarefy* function from version 2.6–2 of the R package vegan. The rarefaction depth of 1000 bp was determined visually using the elbow method for each project by plotting the read depth against the number of samples below that read depth.

### Statistical tests

The Wilcoxon test was calculated using the *wilcoxon* function from version 1.9.1 of the Python library SciPy [26].

### Machine learning algorithm selection

From version 1.1.2 of the scikit-learn Python library, we selected the following MLAs to compare: logistic regression, linear discriminant analysis, k-nearest neighbors, decision tree, random forest classifier, Gaussian naïve Bayes, and support vector machines [27]. The SILVA LTP reference tree transformation with each of PhILR’s 24 weighting scheme combinations was tested with tenfold cross-validation for each of the selected metadata features of each of the datasets.

### Random forest comparisons

To create training and testing datasets, we randomly assigned ¾ of our data to training and ¼ to testing for processing by the random forest algorithm. We employed this shuffle/analysis cycle 20 times for each feature of each dataset.

The random forest models were created using version 1.1.2 of the scikit-learn Python library, and the in-built scoring methods were used to record accuracy [27]. For categorical features, scikit-learn reports the accuracy as the correct number of predictions over the total number of predictions. For numeric data, scikit-learn reports the accuracy score as the coefficient of determination r^2^, defined as (1 − *uv*), where *u* is the residual sum of squares and *v* is the total sum of squares.

## Results

Our test platform consists of 65 metadata features across 4 publicly available datasets. These features include 10 binary categories (such as sex in the Jones dataset), 37 categories with multiple levels (such as ethnicity in the Vangay dataset), and 18 quantitative variables (such as BMI in Jones dataset). For categorical data, we report scikit-learn’s accuracy, and for quantitative variables, we used scikit-learn’s reported *r*^2^ (see methods). We used this platform to evaluate the accuracy of selected compositionally aware and compositionally naïve data transformations. Our compositionally aware transformations included the alr, the clr, and PhILR’s implementation of the ilr. Our compositionally naïve transformations included rarefaction, simple proportions, Hellinger, lognorm, and raw counts tables (see Materials and methods and Background). Our criteria for evaluating the performance of each transformation were simply the *r*^2^ and accuracy scores returned by the random forest for each metadata feature. Each transformation was given the same training and testing sets for each metadata feature, and each metadata feature was given equal weight in our evaluation.

### Random forest is an effective untuned MLA for our data across weighting schemes

Previous literature has suggested that random forest is reliably among the most accurate MLAs for microbiome datasets [4, 28, 29]. To confirm this is the case for our datasets, we compared the random forest classifier with six other algorithms available in Python’s scikit-learn: logistic regression, linear discriminant analysis, k-nearest neighbors, decision tree, Gaussian naïve Bayes, and support vector machines using tenfold cross validation. For PhILR, we also sought to find a suitable weighting scheme for downstream analysis. We tested all 7 of the MLAs with each of the 24 combinations of the PhILR weighting schemes using only our unfiltered SILVA LTP tree from each dataset on their respective categorical metadata. We found that no individual weighting scheme gave consistently good results across all features (Supplementary C). However, consistent with the previous literature, we found that random forest was reliably among the highest performing algorithms and generally gave consistent results across different PhILR weighting schemes. We chose to reduce the scope of downstream analyses by only considering random forest and the “blw.sqrt” (branch length weight square root) for phylogenetic distance weighting and “enorm” (Euclidean norm) for taxon weighing. To include quantitative data in our evaluation, for downstream analysis, we also used random forest regressor for quantitative features.

### Proportion-based transformations have the highest average accuracy across all metadata categories

**Fig. 3:**
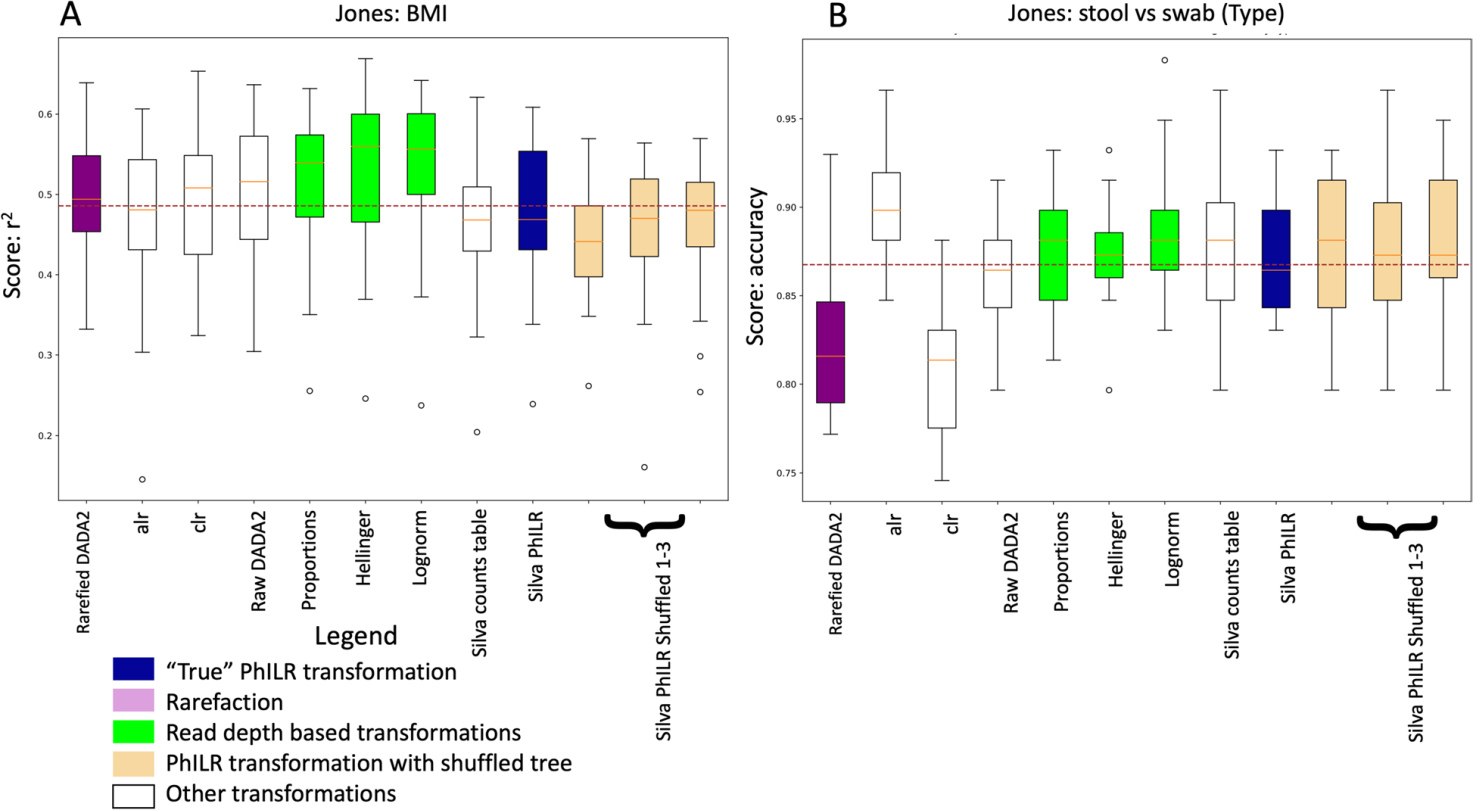
Typical box and whiskers plots of random forest scores for single features and selected transformations. This plot shows the scores of the random forest regressions and classifiers on BMI (**A**) and “stool vs swab” (**B**) in the Jones dataset, respectively. The *x*-axis shows each transformation and the *y*-axis shows the score, which was *r*^2^ for the quantitative BMI and accuracy for the categorical stool vs swab (type). For the sake of simplicity, only the transformations made from the unfiltered counts table are shown and the Silva/LTP PhILR transformations are shown as the lone representative of the various PhILR transformations we examined

Having selected the random forest classifier and regressors as our MLA and blw.sqrt and enorm as our PhILR weighting schemes for downstream analysis, we tested each transformation against each of the 65 metadata features of our datasets. For each transformation and each metadata category, we performed 20 iterations randomly dividing the data into 75% of the data for training and 25% for testing. This created 20 accuracy scores for each transformation and each metadata feature. We repeated this analysis twice, once where we created transformations starting with an unfiltered counts table and then again where we started with a prevalence filtered counts table that removed features that were not found in at least 10% of the samples. The exception to this was the raw count-based UPGMA and IQTREE PhILR transformations, which required filtering before tree building, as the number of sequences was more than the R packages could handle. For these, we used a high variance/low abundance filter. As controls to our PhILR transformations, we created 3 random shuffles of the nodes of each tree and made PhILR transformations with them. The results of these analyses are captured as 65 sets of boxplots, each with 49 transformations. However, to reduce the clutter in our figures, we chose to show only the highest performing PhILR transformation for each filtering type, which was the Silva PhILR. As an example, we consider *r*^2^ for a BMI and accuracy from “stool vs swab” from the unfiltered counts table-based Jones datasets (Fig. 3). We see that the lognorm transformations in this example (green bars) have a higher *r*^2^ than most of the other transformations.

**Fig. 4:**
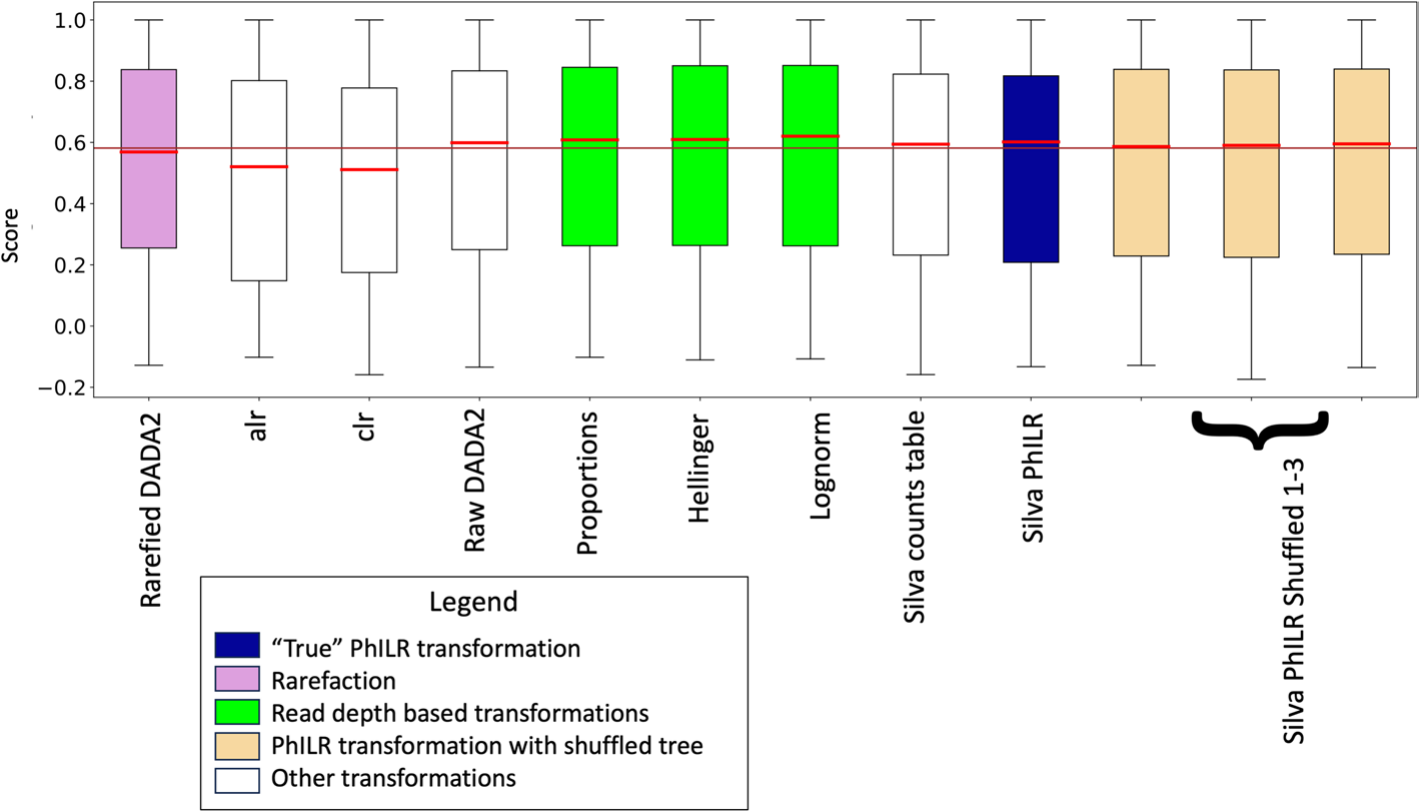
For unfiltered tables, proportion-based transformations have the highest accuracy. These box and whisker plots show the average of all the points in each metadata feature for each transformation (65 points for each transformation). The red bars represent the median for each transformation and the brown line represents the median of the entire dataset platform

To summarize performance across all 49 transformations, we averaged each transformation across all of the 65 metadata features. When looking at the transformations that started from an unfiltered table, the proportion-based transformations such as proportions, Hellinger, and lognorm on average yield a small improvement when compared to every other transformation (Fig. 4). For the transformations that started from a prevalence filtered counts table, the proportion-based transformations also have the highest median accuracy (Supplementary Figure D). Surprisingly, the alr and the clr have noticeably worse average scores for both filtering strategies.

**Fig. 5:**
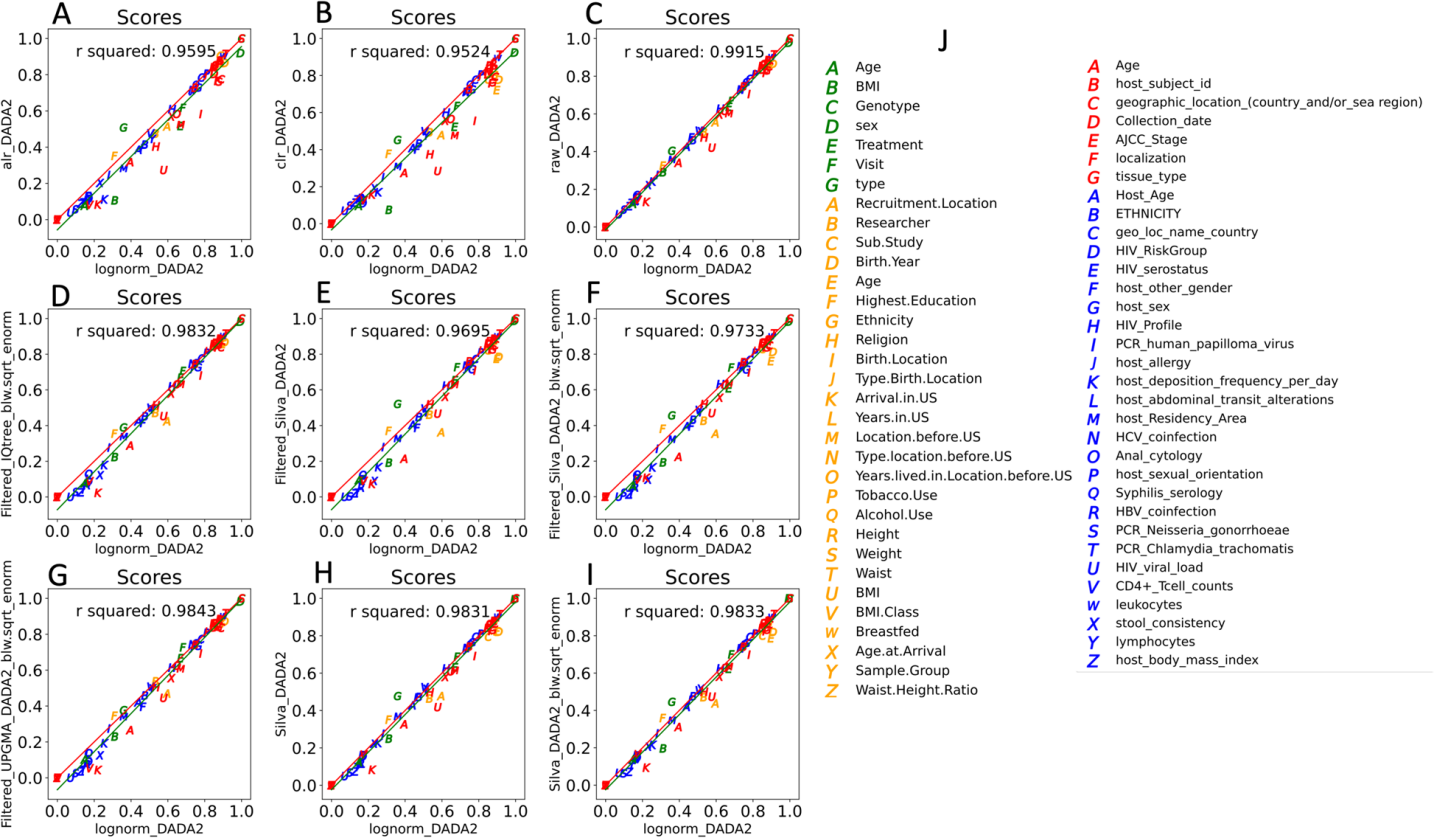
Accuracy vs accuracy plots show how lognorm provides better accuracy for random forest than non-proportion-based transformations. Points for each plot represent scores of random forest classifier (accuracy) and random forest regressor (*r*^2^). Lognorm of raw DADA2 counts tables performs favorably compared to filtered alr (**A**), clr (**B**), raw DADA2 (**C**), filtered IQTREE PhILR (**D**), filtered SILVA DADA2 counts table (**E**), filtered SILVA DADA2 PhILR (**F**), filtered UPGMA PhILR (**G**), SILVA DADA2 counts table (**H**), and SILVA DADA2 PhILR (**I**). Each metadata feature of each dataset is shown in **K**

To begin to address the statistical significance of the differences between different transformations, we compared all 49 transformations pairwise to each other by creating accuracy vs accuracy plots. To do this, we plotted the average score for each metadata feature for a given transformation against the average score of each metadata for another transformation—for this, we included both the accuracy and *r*^2^. This yields 1176 (49 choose 2) pairwise comparisons. In general, different transformations yielded highly similar performance: among the 1176 separate pairwise comparisons, *r*^2^ values ranged from 0.94 to 0.9997 and had a median of 0.986. In general, across different choices of PhILR inputs including shuffled trees, and the different methods for making phylogenetic trees to feed into PhILR, these transformations made little difference to performance and yielded scatter plots of accuracy with high *r*^2^ values. However, we noticed that scatter plots involving the proportion-based transformations showed a small but consistent improvement across most of the 65 metadata categories when compared to the other transformations. Lognorm exemplifies the small improvements over other types of transformations (Fig. 5).

**Fig. 6:**
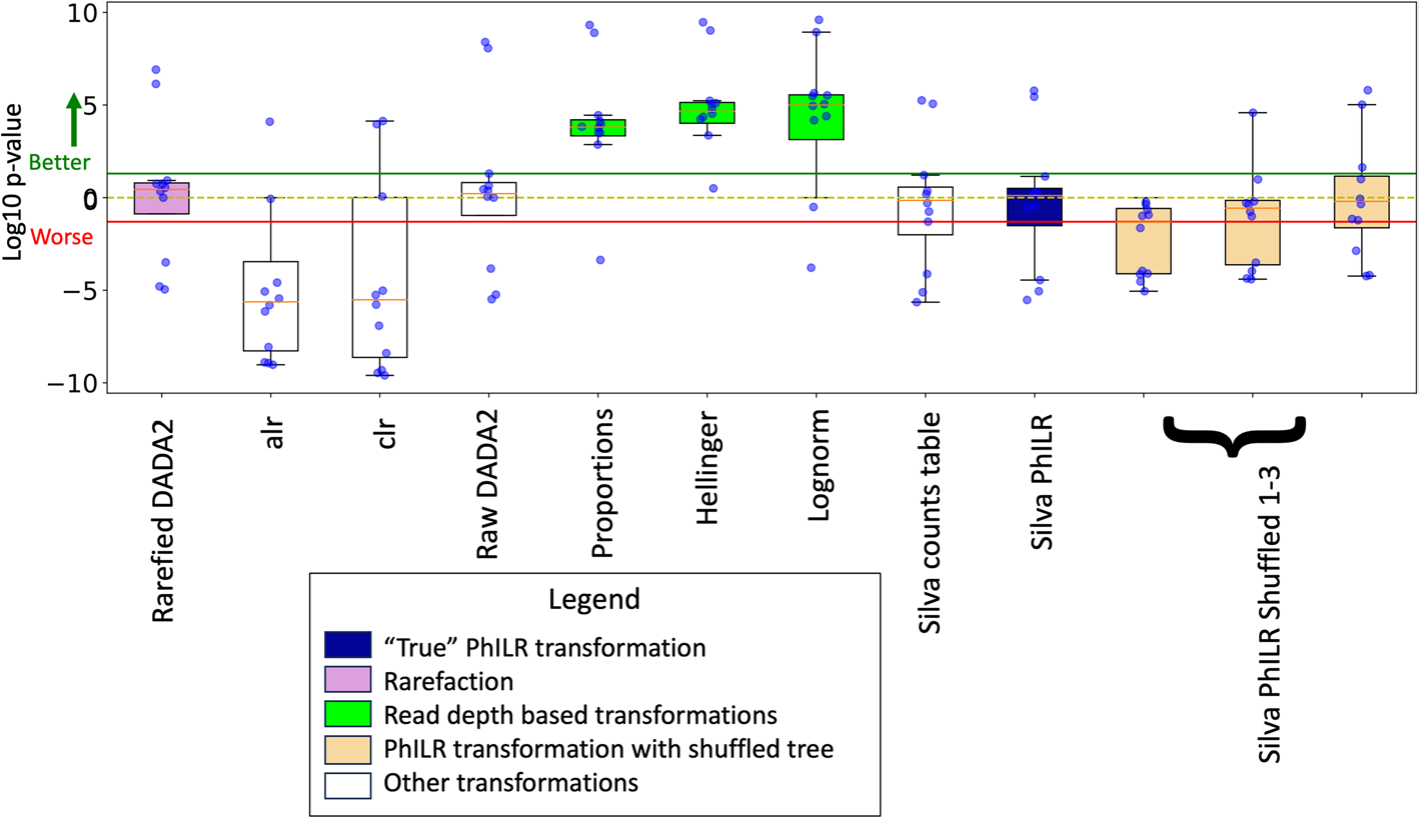
Pairwise *p*-values indicate that lognorm performs significantly better than every other transformation. The *y*-axis shows log10 of the *p*-value, and the *x*-axis shows how well each transformation performed against the other 11 transformations. A positive value indicates that the average accuracy of the given transformation is higher than others, and a negative *p*-value indicates that the average accuracy is lower than the others. The area above the solid green line represents the sample space where the transformation is significantly better, and the area below the solid red line represents the area where points are significantly worse

In order to assess patterns of statistical significance and compare the small effect-sizes, we next calculated pairwise Wilcoxon *p*-value across each of the 65 metadata variables comparing each of the selected transformation against the other 48 transformations. In order to record which transformations performed better at each Wilcoxon test, we recorded the *p*-value with a positive sign if the selected transformation performed better and a negative sign if it performed worse. This allowed us to plot the significance and the performance of each transformation. For the transformations (Fig. 6), we assessed a null hypothesis that each selected transformation has similar performance to the other normalization schemes. From this, we can see that proportion-based transformations are clearly outperforming the other transformation and that the alr and clr are among the worst performing transformations. Furthermore, we can see that rarefaction provides no advantage and that normalizing to read depth as is done by proportions, Hellinger, and lognorm provides the highest accuracy for unfiltered data. When compared to each other, proportion-based transformations do not show any significant difference, and the filtering does not provide a great benefit (Supplementary Figure A4).

## Discussion

Our chosen platform for testing consisted of 65 metadata categories from 4 publicly available datasets. These four datasets included various metadata categories that varied from demographics, diet information, and sexual histories to a variety of disease states and case/control statuses (Table 1). Many of these metadata categories within each dataset are correlated with each other, and we did not explore methods to remove these potential redundancies in the datasets. In our visualizations, we combined information from quantitative variables (such as BMI) and categorical variables (such as case/control) by utilizing scikit-learn’s reported *r*^2^ accuracy value for quantitative variable and simple accuracy for categorical variables. In general, the numeric features where random forest regression was used had lower scores than ones where random forest classification was used, but since we compared all 49 normalizations schemes across the same set of 65 categories, these differences do not bias our results.

Overall, we tested 49 transformations 20 times against the 65 metadata categories for a total of 63,700 random forest trials. We believe that our study represents the one of the most exhaustive analysis of normalization transformations in terms of both the number of schemes considered and the number of metadata categories. Our metadata categories spanned a wide range of effect sizes with some categories that allowed for nearly perfect classification (such as classifying a geographic location of a sample in the Zeller dataset) to others where accuracy was near zero (such as BMI in the Noguera-Julian dataset). This range of effect sizes is a strength of our study as it allows us to evaluate the performance of different normalization schemes across a wide range of signal/noise ratios. One of the interesting results of our study was that differences between normalization schemes were only apparent at intermediate effects sizes (Fig. 5). This makes intuitive sense as for features with large effect sizes, all the MLAs can effectively classify regardless of the small differences produced by different normalization schemes, while for very small effect sizes, there is essentially no signal for the MLAs to latch onto, so normalization transformations make little difference. Plots in Fig. 5 tend to have a bulge of data points near the center of the plot. Thus, a greater variance in the scores occurs where the signal is intermediate—not at the top where the features with the strong signals nor at the bottom where features with a weak signal. Considerations of the strength of the signal are therefore important but have not consistently been explicitly considered in previous studies.

We began our assessment of MLAs by comparing frequently used MLA toolkits including random forest, logistic regression, k-nearest neighbor, and support vector machines. Consistent with previous literature [4, 28, 29], we found that random forest was reliably among the best performing algorithms, and we therefore chose it as our base algorithm for testing. We also assessed the best weighting scheme for PhILR input and found that the choice of weighting scheme made little overall difference to MLA performance. This is consistent with the observation made by Silverman et. al. that random forest is robust to the PhILR transform [10]. In order to limit the scope of our downstream experiments, we chose blw.sqrt and enorm as our two weighting schemes.

In terms of which compositional transformation is best, we found that the PhILR transform is an improvement on the alr and clr but still underperforms transformations that normalize to read depth for use with random forest (Fig. 4). PhILR is also sensitive to the filtering techniques used on its input. We noted that the median score for each “true” PhILR transform in Fig. 5 was either about as good as or slightly higher than the median score for the counts table from which it was made. This indicates that the PhILR transform may improve the counts tables from which it is directly made or at least will not cause worse performance. Our results using ASVs may differ from those using OTUs or those using different filtering techniques.

Somewhat surprisingly, our results indicate that the quality of trees used for the PhILR transform do not matter for either the ilr transformation or the weighting schemes, as no “true” tree consistently outperformed the series of randomly generated trees in which nodes were joined independently of actual sequence. We had similar results wth UPGMA and IQTEE2 for each different filtering method. The PhILR transformations that come from the high variance and low abundance filtered datasets all gave similar performance despite the differences in the trees. We conclude that the quality of the tree used for a transformation such as PhILR and the weighting scheme used are much less important for MLA than the quality of the counts table.

There are a very large number of possible unique combinations of preprocessing steps, transformations, and MLAs that could potentially be used in microbiome studies, and we cannot test all possible combinations. This inevitably leads to potentially important transformations that we did not explore. For example, there are as many alr transformations as there are taxonomic features or ASVs in each dataset. There are therefore many more alr’s available than the one that we tested, which was simply using the taxa with the fewest zero entries as the reference taxa in the denominator. While we found that this choice for alr was our worst overall performing transformation, it is possible that future work could better tune the alr. This space has been explored by others who have suggested that variance and covariance and Procrustes analysis can aid in finding the best reference taxa [30]. Another option that we did not explore with these datasets was to use a spike-in control sequence of known abundance as our denominator [31]. We believe that this method would have likely improved the performance of the alr.

Another limitation of our study is that we have included only gut microbiome datasets in our sample data. Our choice here reflects the broad interest in the scientific community in using the gut microbiome to predict the occurrence of inflammation-mediated diseases. While there is no reason to think that our observations would not extend to other microbial environments, this will need to be directly established by future research. Finally, we also only looked at classification at the ASV level. A previous study has argued that ASVs generally provide better resolution than OTUs for 16S gene sequencing experiments [3], although this view is not universally shared [1].

## Conclusions

Our study suggests that simple compositionally naïve transformations such as log-normalization or even not normalizing data at all can outperform more sophisticated compositionally aware transformations in machine learning applications in the human gut microbiome. A straightforward interpretation of our results is that the random forest algorithm is robust to many artifacts in microbial sequence data, and therefore, the simplest possible transformations to the data that incorporate sequence depth information, such as our lognorm transformation, give the random forest algorithm the clearest view of the classification task to be achieved. Future studies will determine if these observations are general and can be applied to other microbial environments and other genomic datasets such as transcriptomics and GWAS studies.

### Supplementary Information

**Additional file 1: Supplementary A. **Images of “true” trees. Images of the unfiltered SILVA LTP tree, the high variance/low abundance filtered SILVA LTP tree, the UPGMA, and IQTREE for each dataset (Vangay, Jones, Noguera-Julian, and Zeller).

**Additional file 2: Supplementary B. **Schematic representation of workflow for the creation of data transformations from the prevalence filtered Dada2 counts tables. This schematic starts with the prevalence filtered Dada2 counts tables and each step in the workflow (grey arrows with white bubbles) lead to trees (grey blocks) or final datasets (blue boxes) as described in the methods section. Datasets with random node shuffles were recreated at least 3 times.

**Additional file 3: Supplementary C. **Average scores of MLAs across PhILR weights from the Silva/LTP PhILR made from the raw counts table. To help determine which MLA to use for this project, we used 10-fold cross-validation to train and test each MLA on each PhILR weighting combination of for each of the 65 features. Within each dataset, for each PhILR combination, the means of all the features from each MLA (y-axis) were plotted showing the performance of each metadata feature for each PhILR weighting combination (x-axis).

**Additional file 4: Supplementary D. **For prevalence filtered tables, proportion-based transformations have the highest median accuracy. These box and whisker plots show the average of all the points in each metadata feature for each transformation (65 points for each transformation). The red bars represent the median for each transformation and the brown line represents the median of the entire dataset platform.

## Data Availability

All shell, Python, and R scripts and dataset metadata used for this project are available in the Git repository: https://github.com/amyerke/lognorm_vs_CODA. All datasets are publicly available at the following locations: **Table:** 

Name, reference	Accession number and link
Vangay [13]	PRJEB28687
Jones [17]	PRJNA397450
Zeller [18]	PRJNA397450
Noguera-Julian [19]	PRJNA307231

## Abbreviations

Alr: Additive log ratio
Clr: Centered log ratio
Ilr: Isometric log ratio
ASV: Amplicon sequence variant
AUC: Area under the curve
MLA: Machine learning algorithm
PCA: Principal components analysis
GWAS: Genome Wide Association Study
