# Supplementary material for: Proportion-based normalizations outperform compositional data transformations in machine learning applications
Source: Microbiome. 2024 Mar 5;12:45. doi: 10.1186/s40168-023-01747-z (PMC10913632; doi:10.1186/s40168-023-01747-z)

orig_ref

Zeller Silva LTP

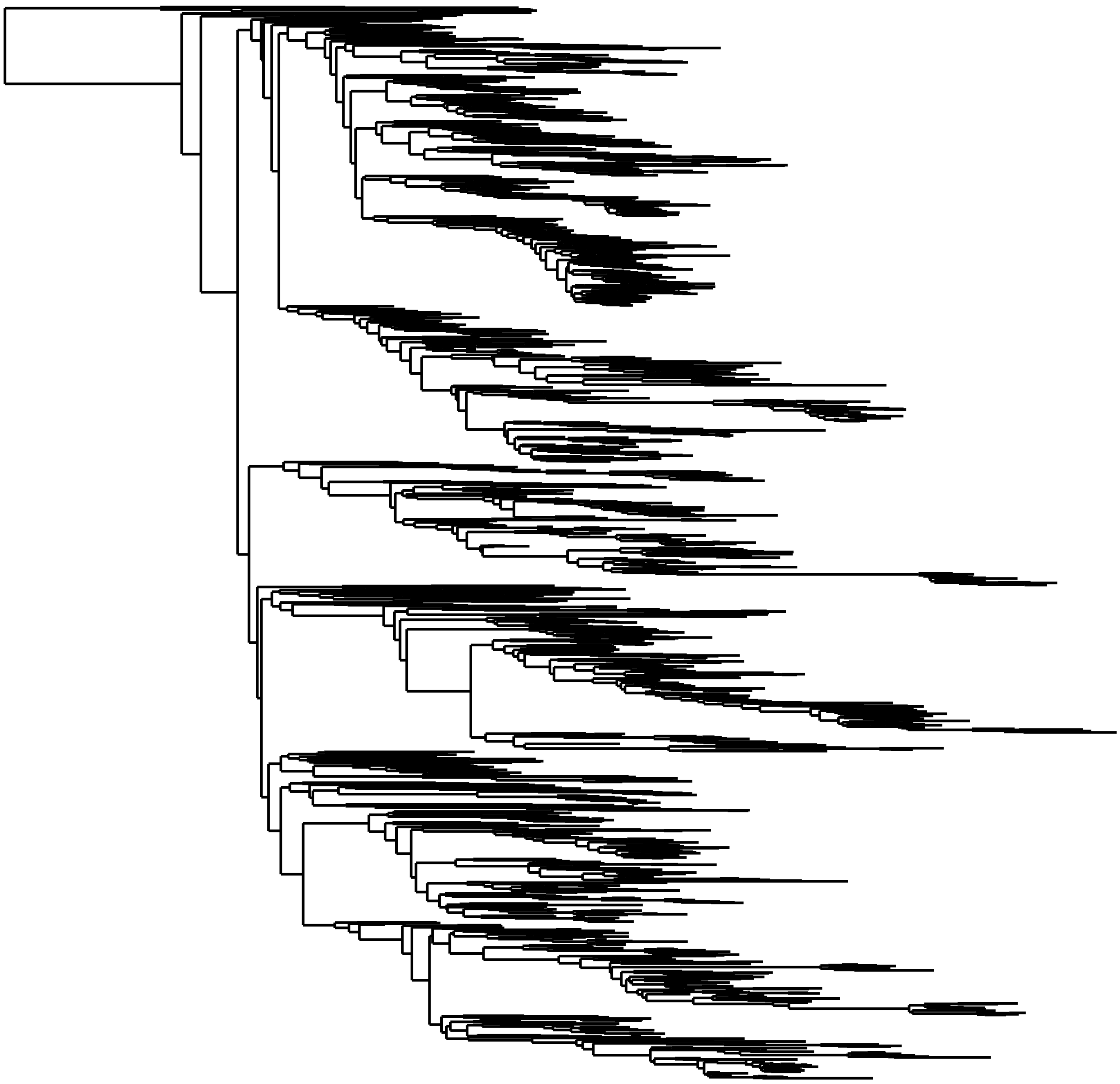

cln_ref

Zeller filtered Silva LTP

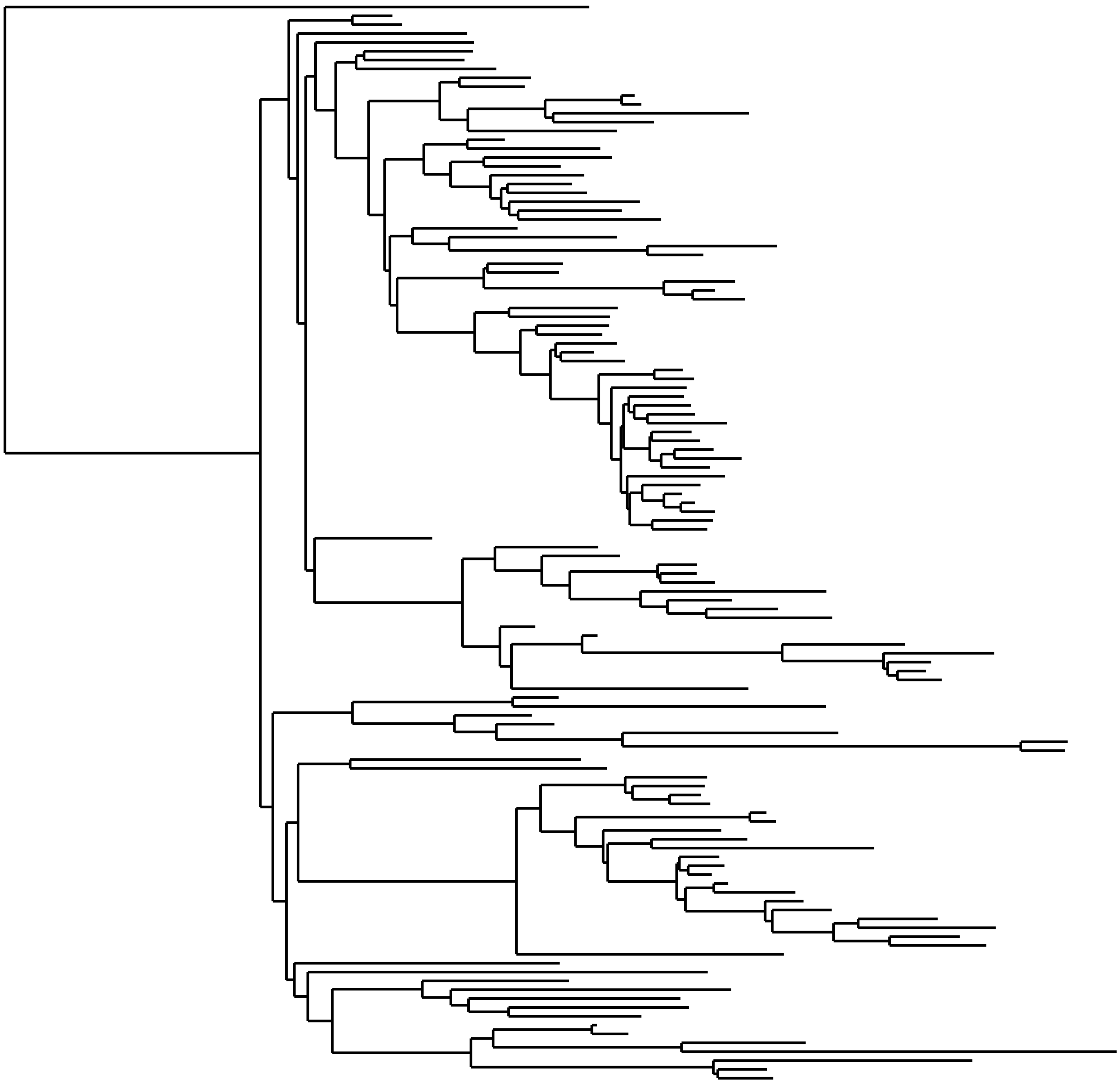

orig_upgma

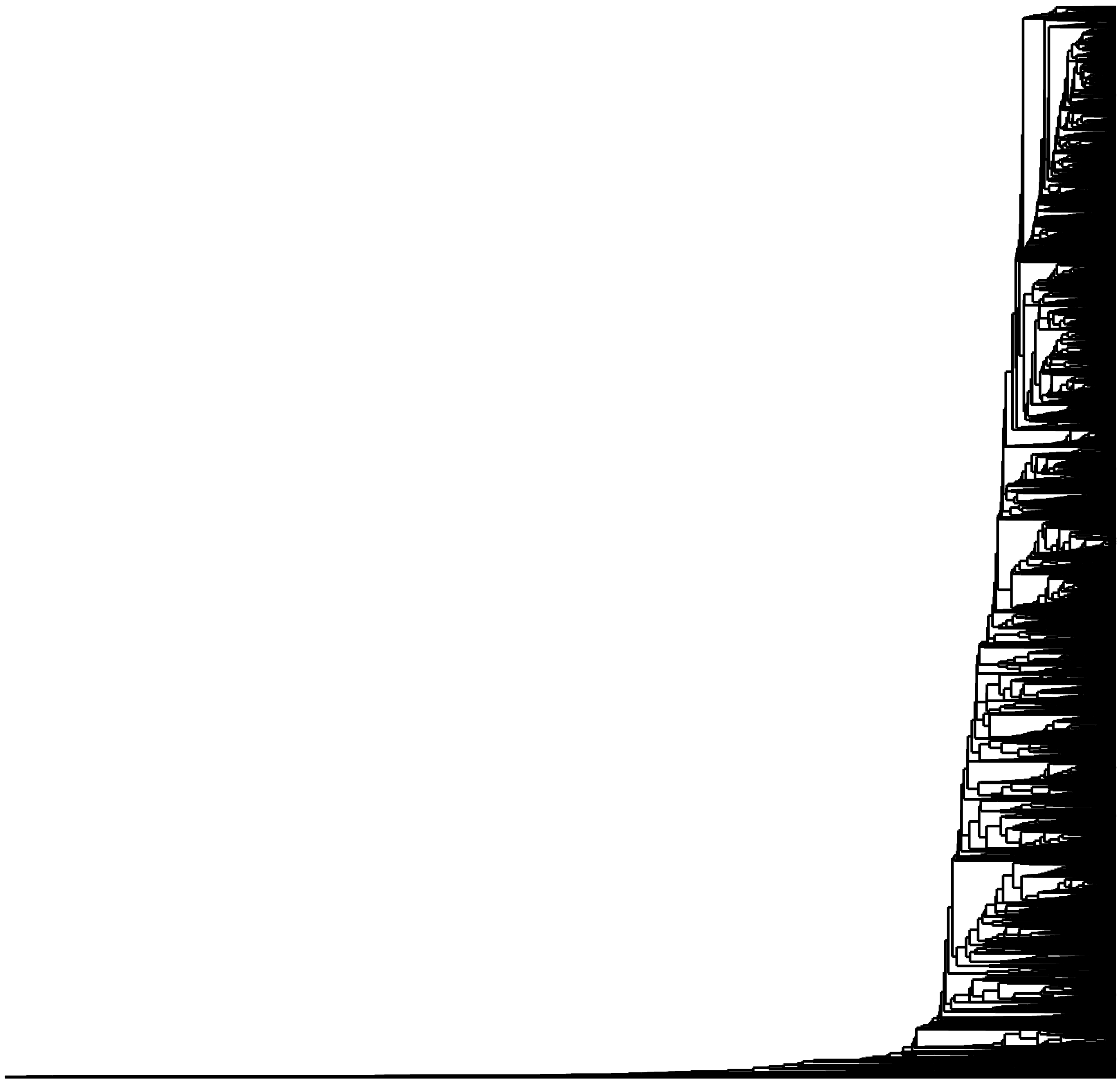

cln_upgma

Zeller Filtered UPGMA

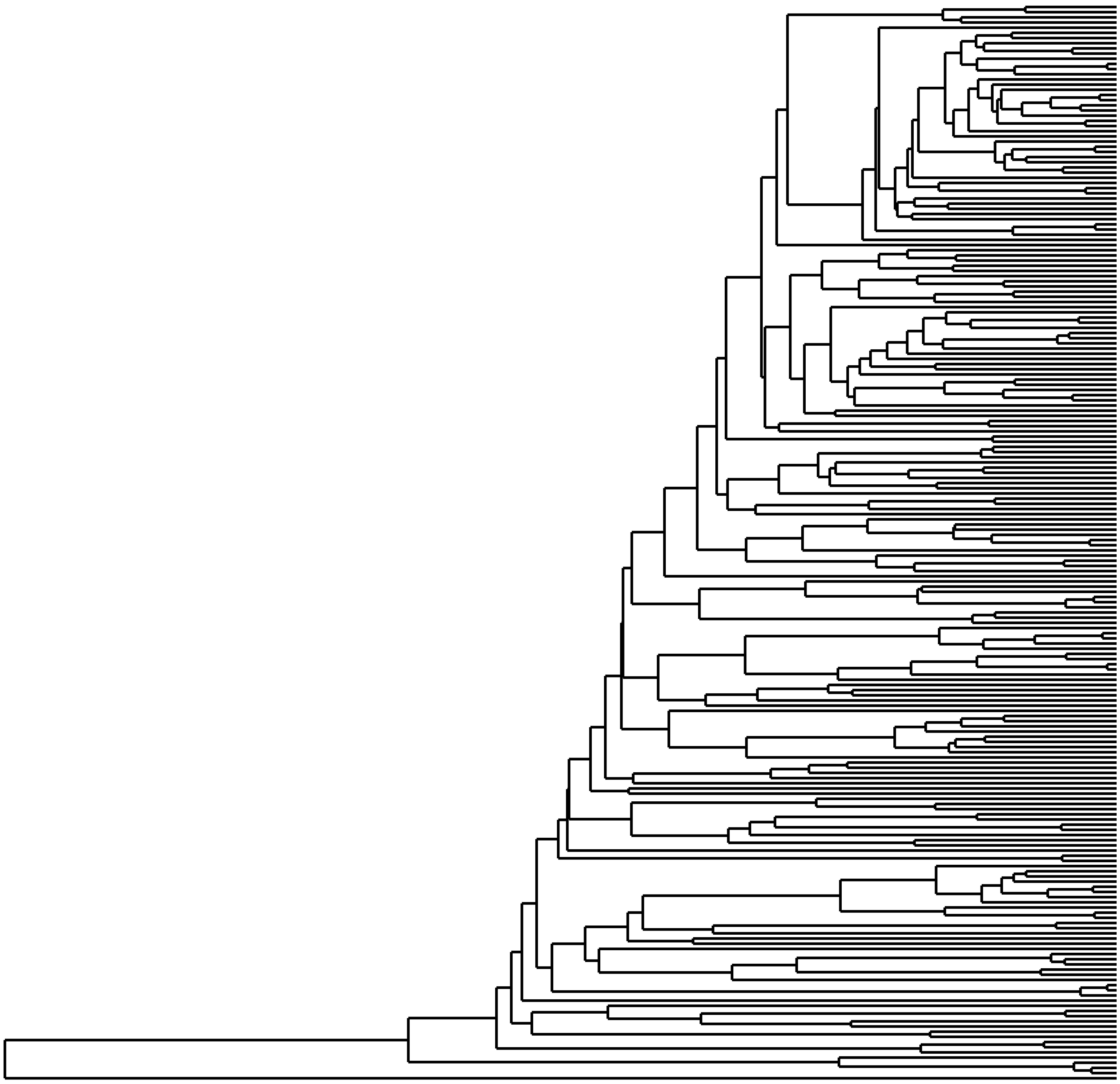

orig_iqtree

Zeller IQTREE

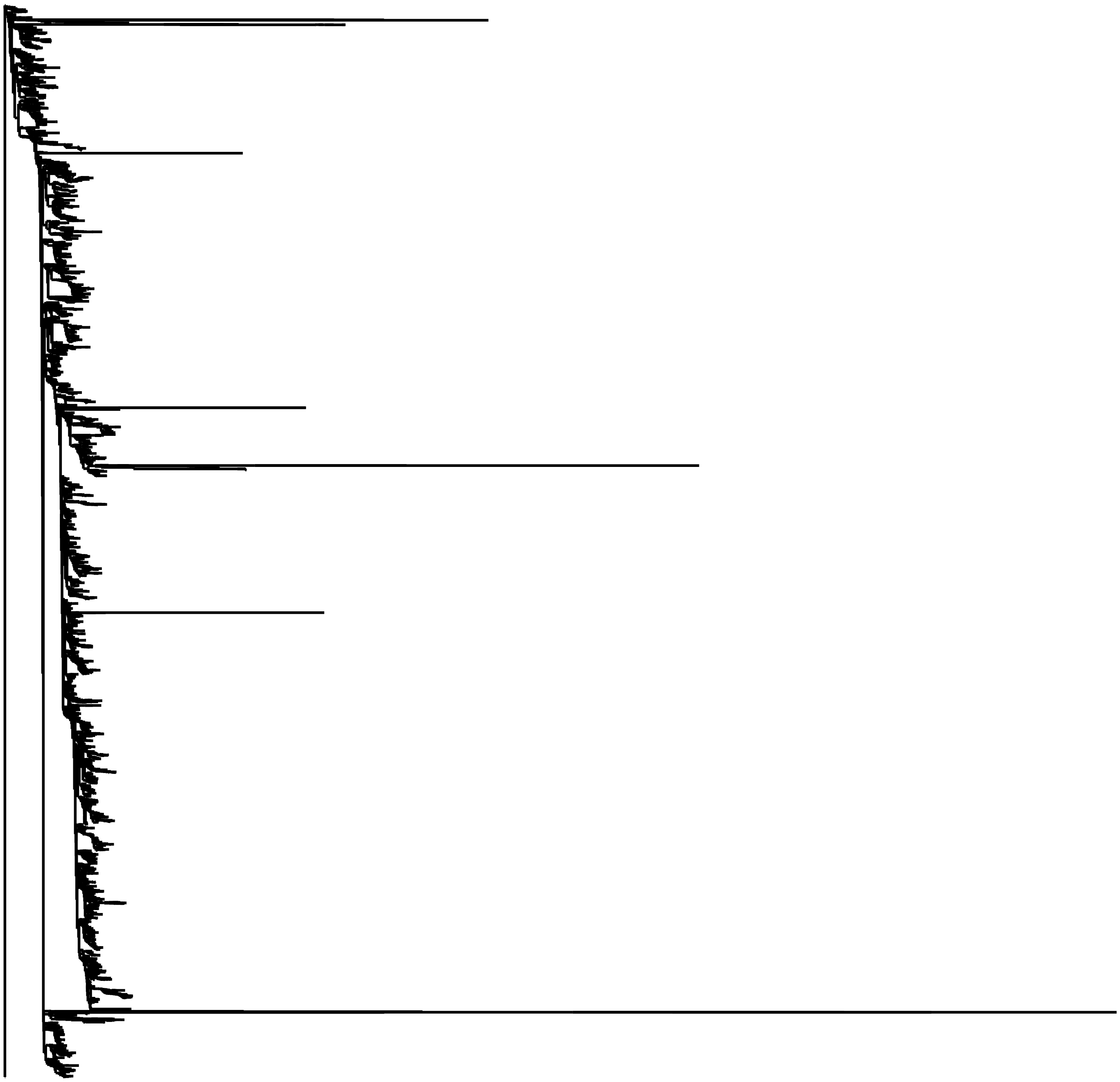

cln_iqtree

Zeller filtered IQTREE

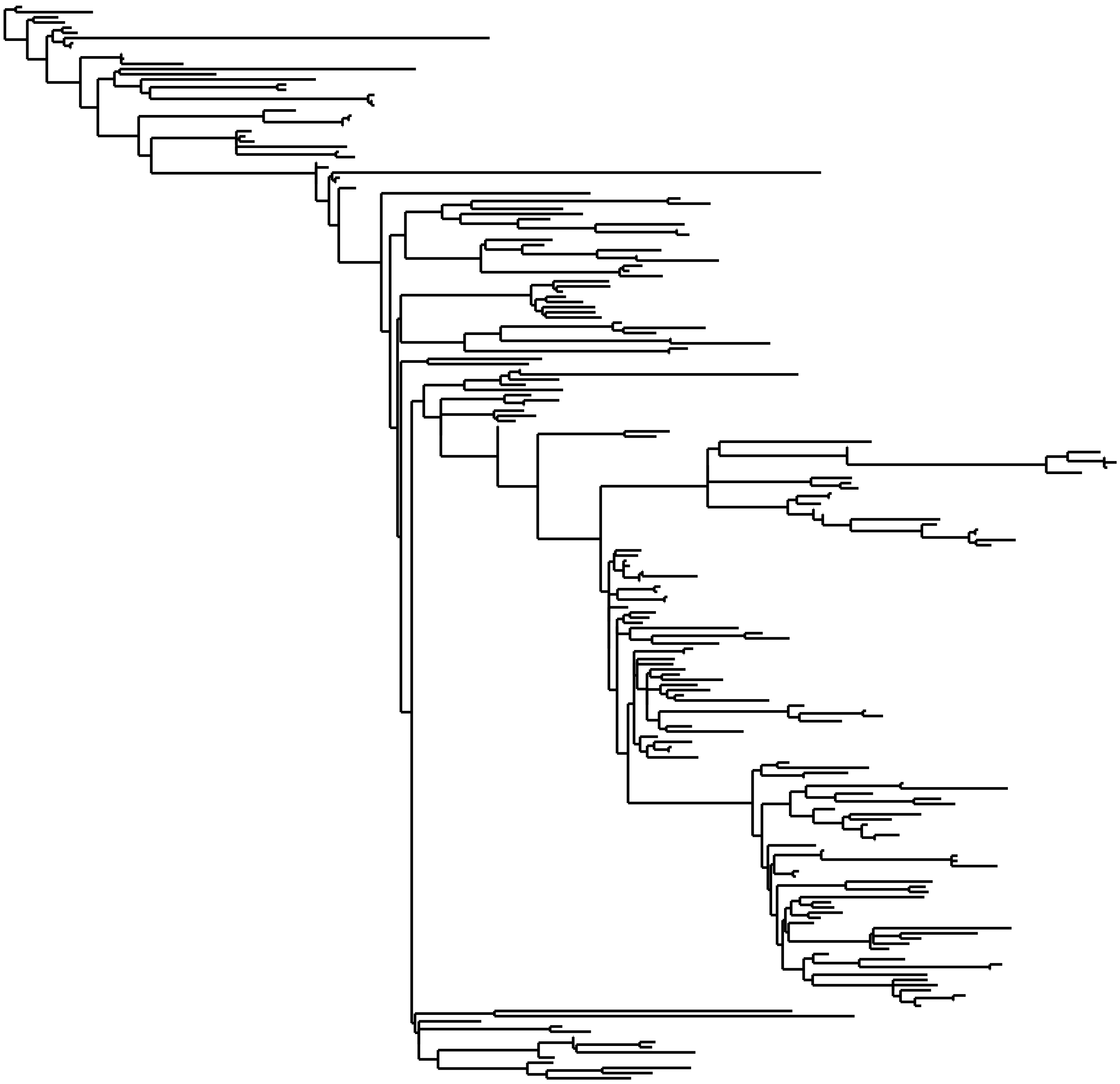

orig_ref

Jones Silva LTP

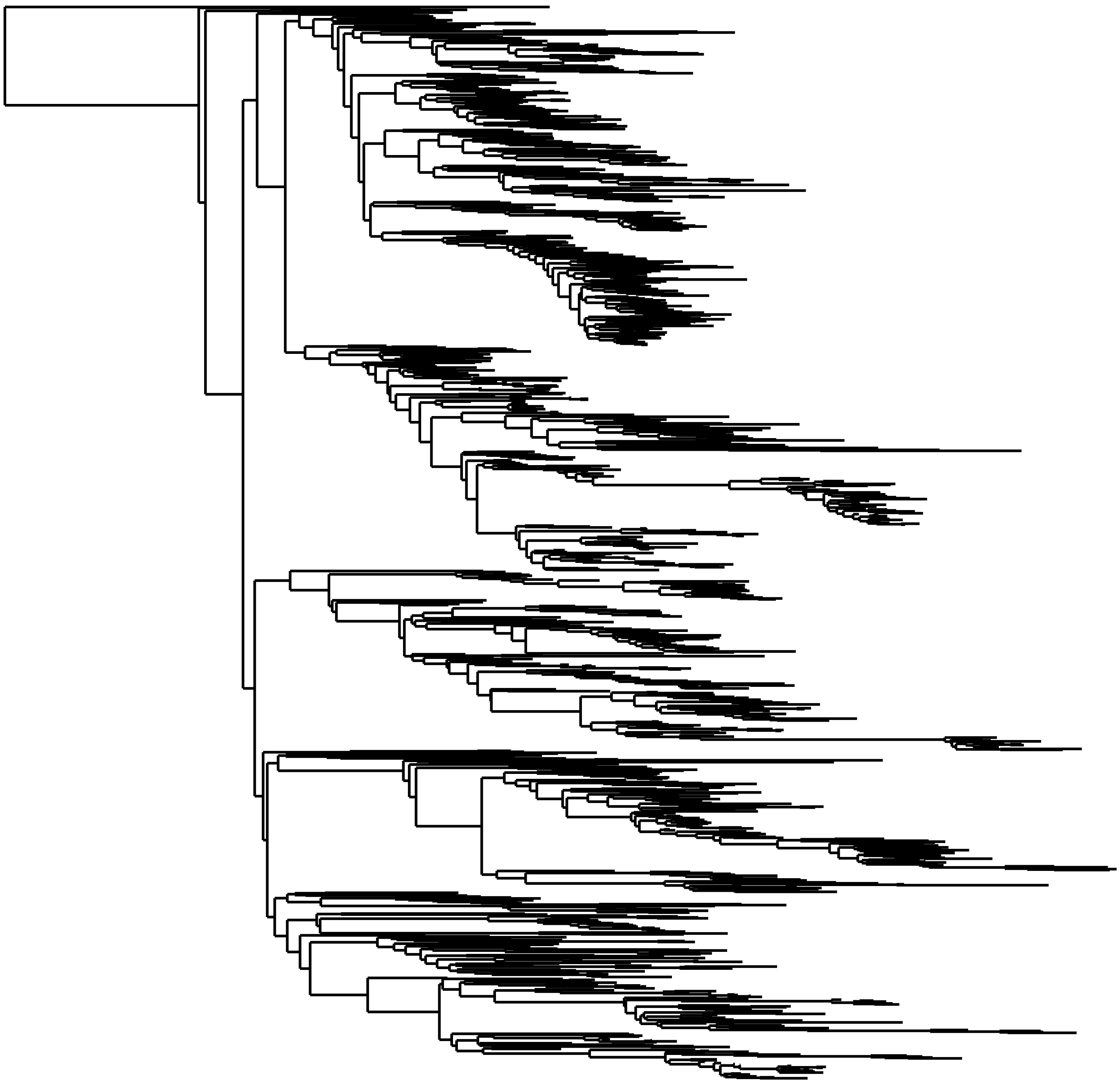

cln_ref

Jones filtered Silva LTP

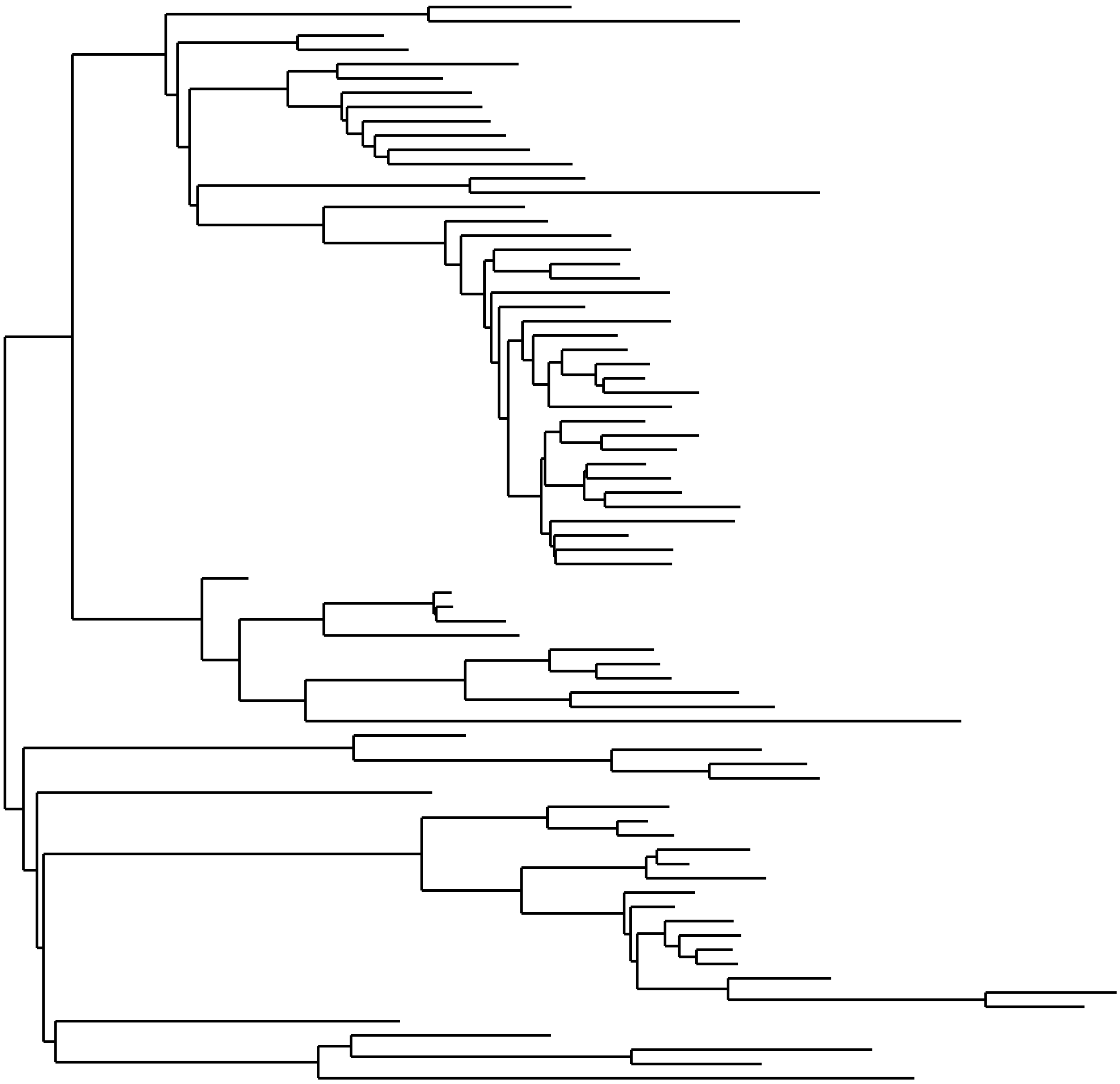

orig_upgma

Jones UPGMA

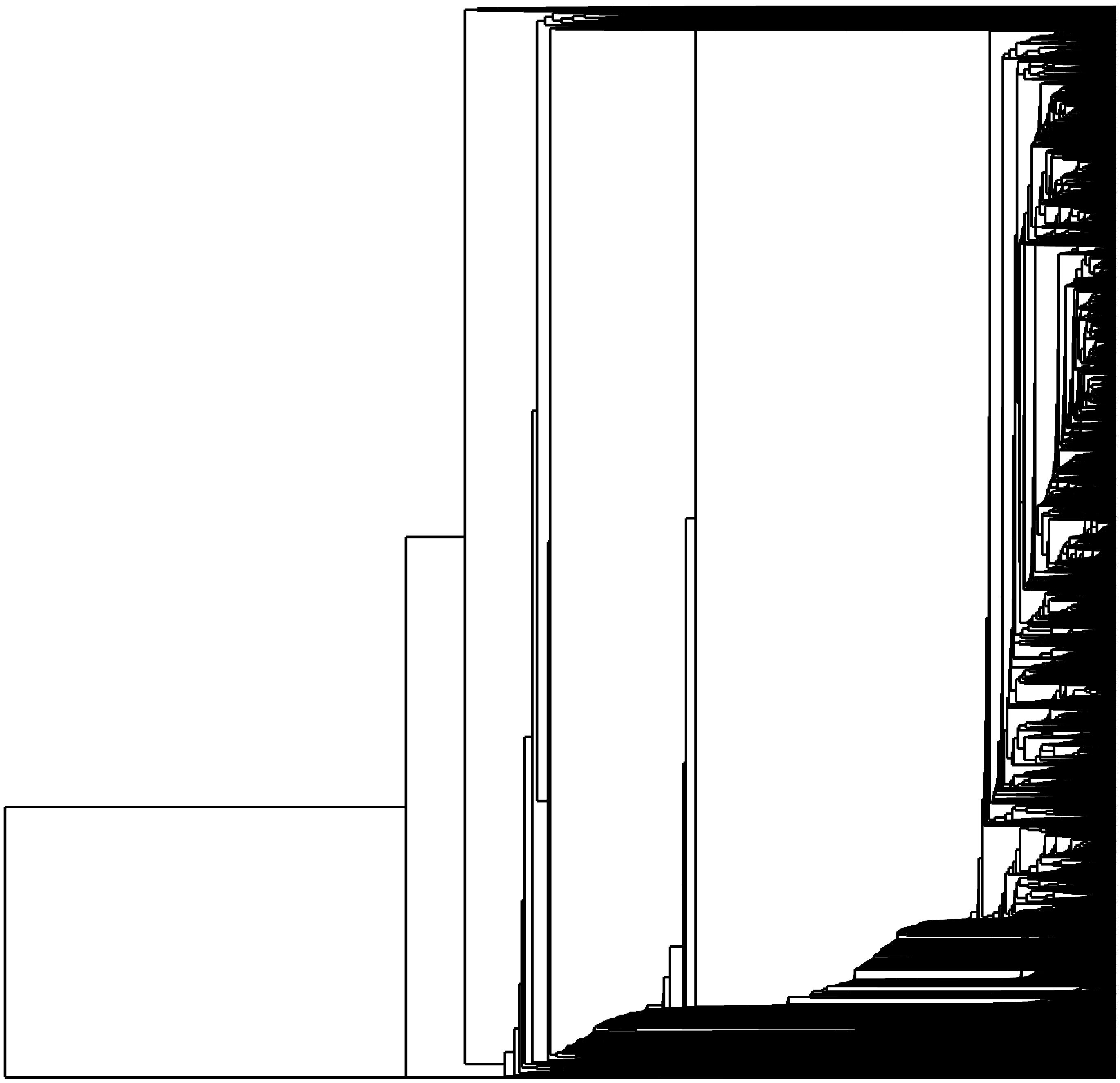

cln_upgma

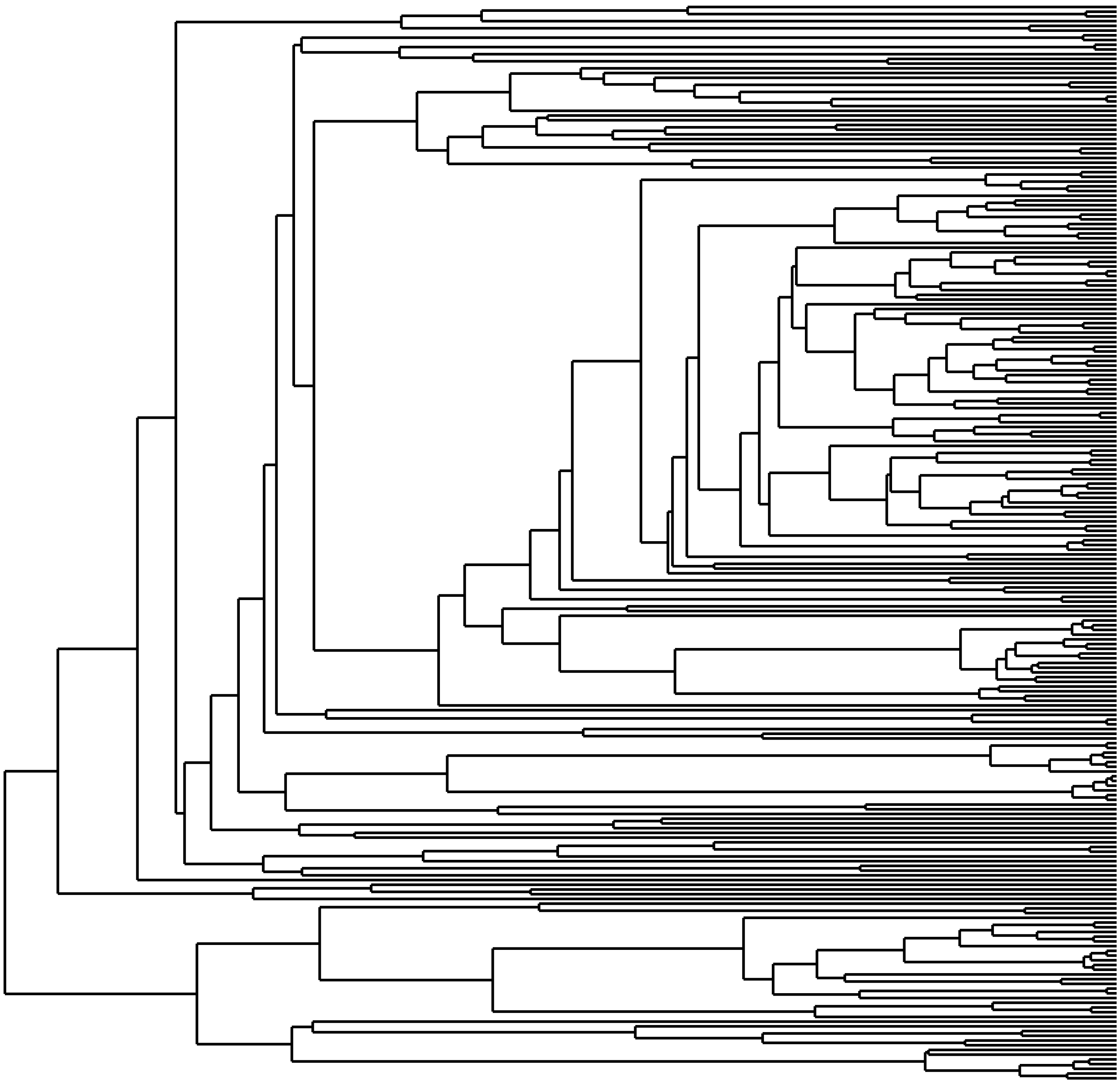

orig_iqtree

Jones IQTREE

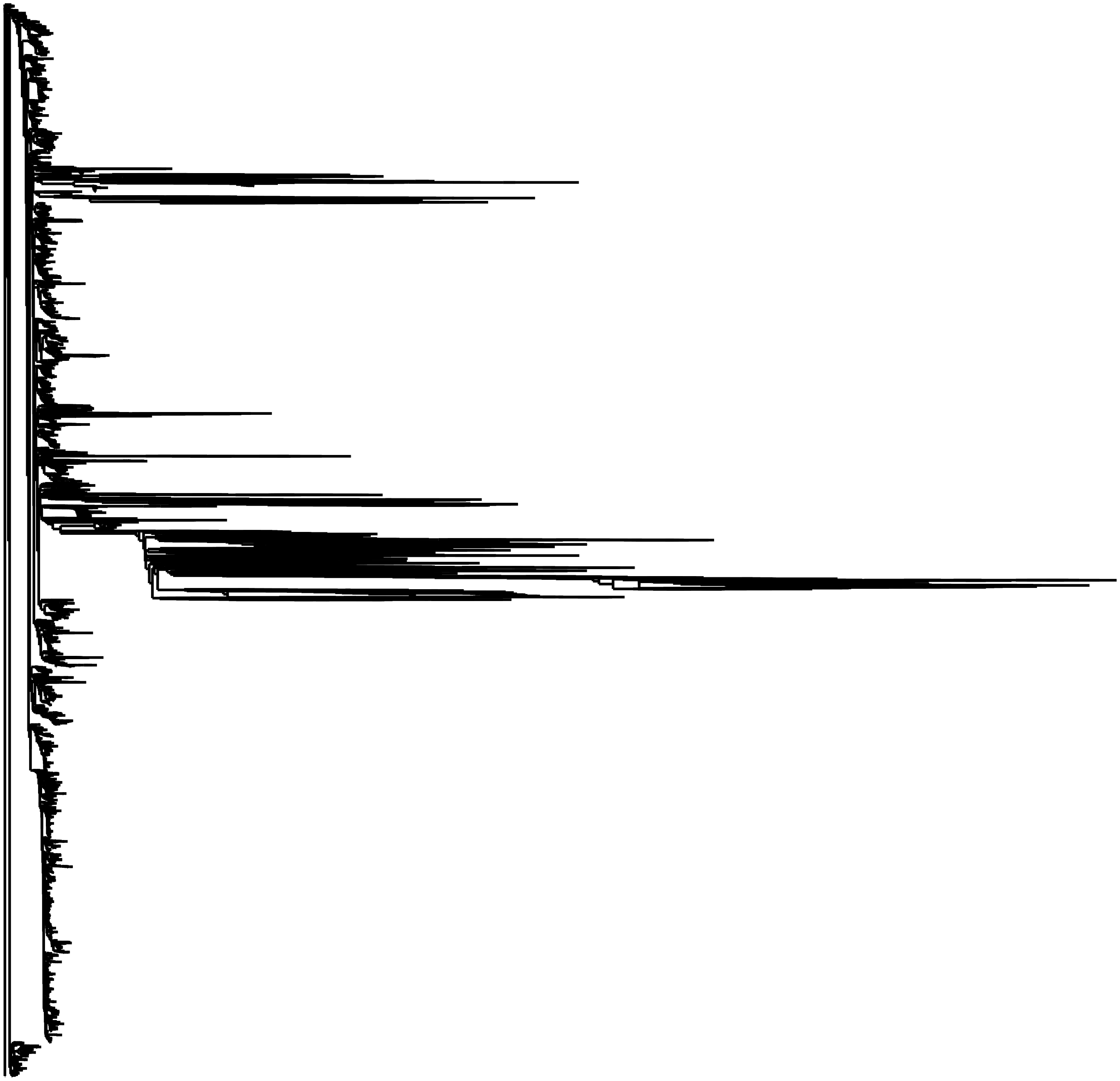

cln_iqtree

Jones filtered IQTREE

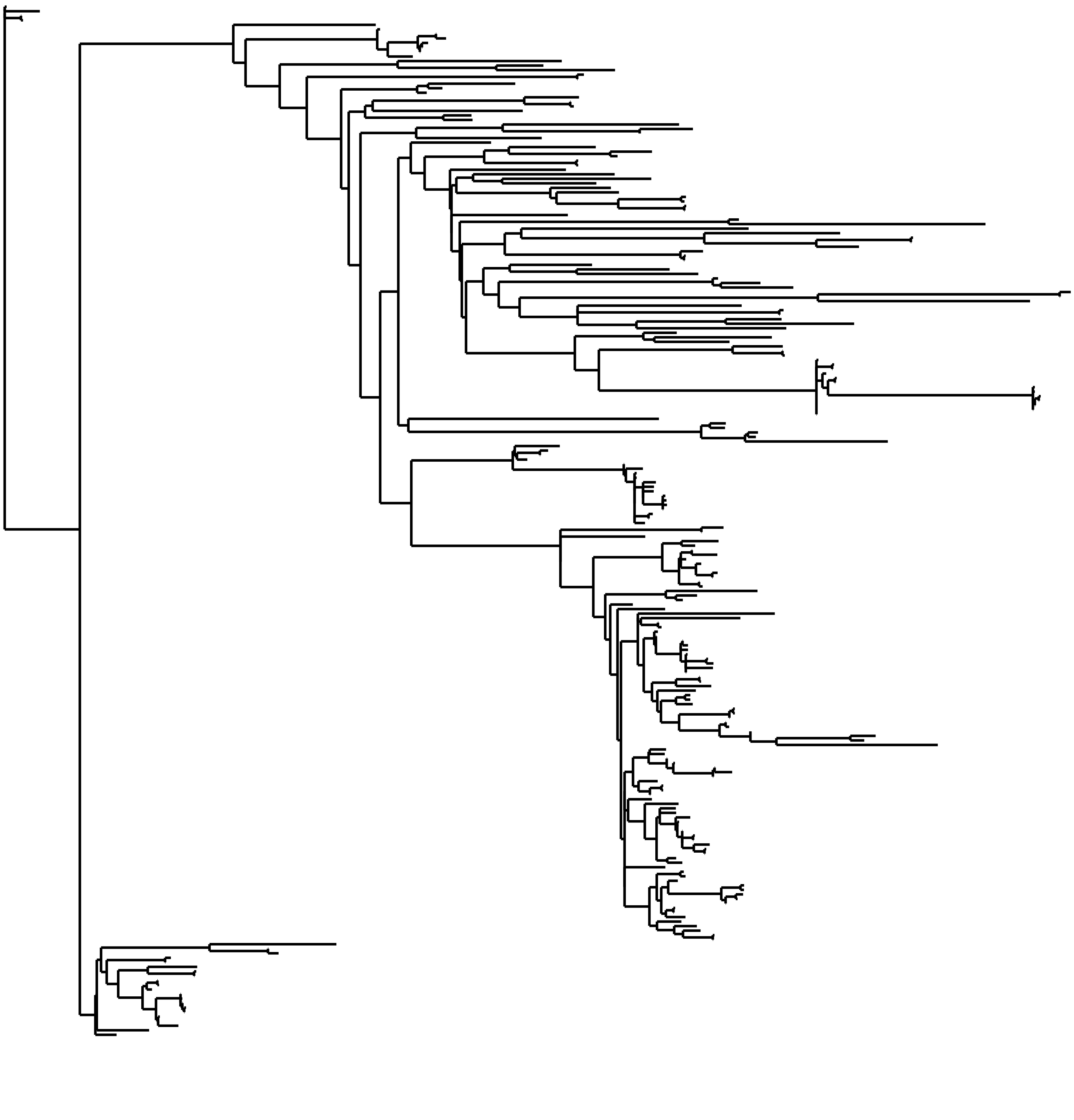

orig_ref

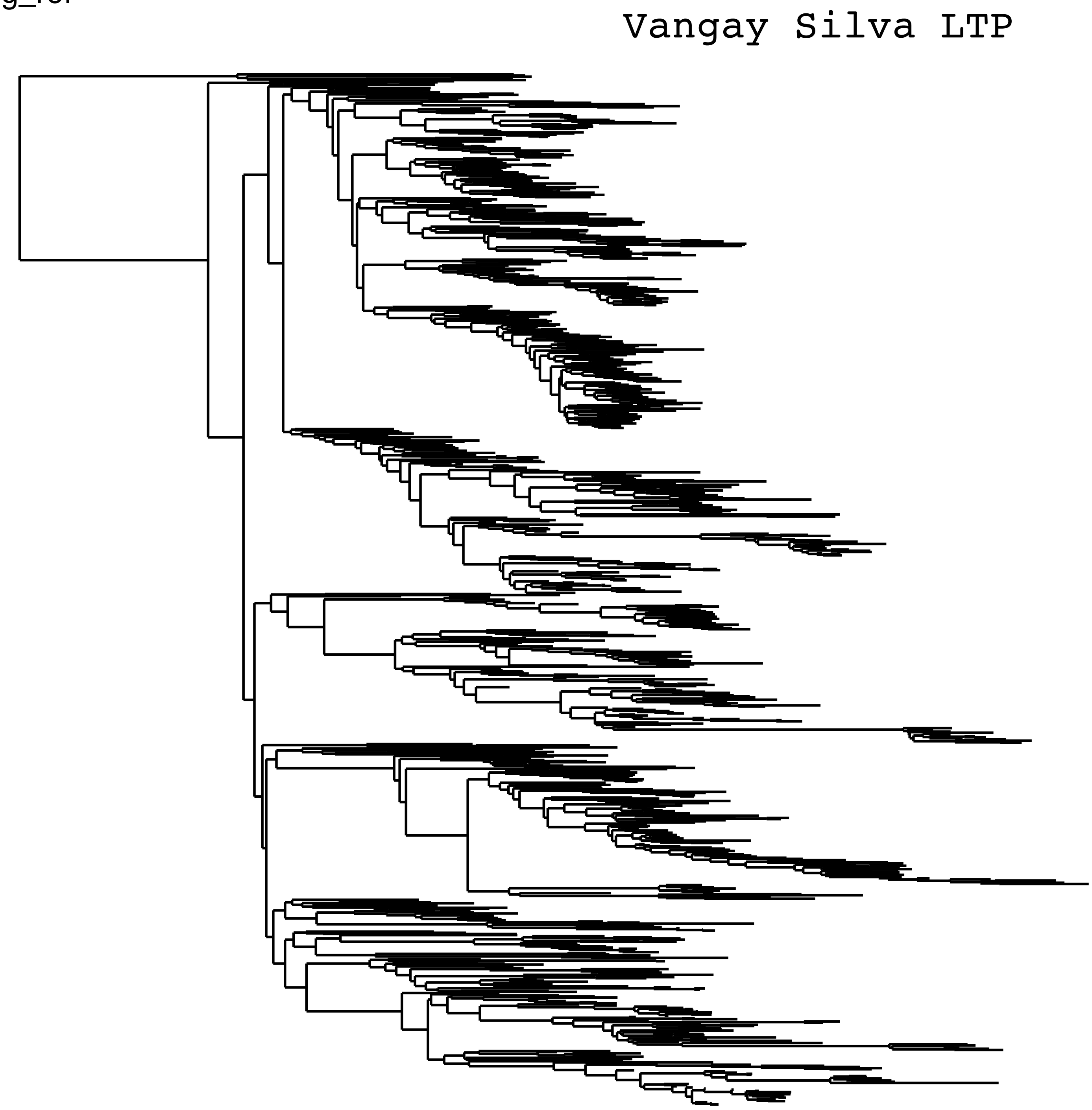

cln_ref

Vangay filtered Silva LTP

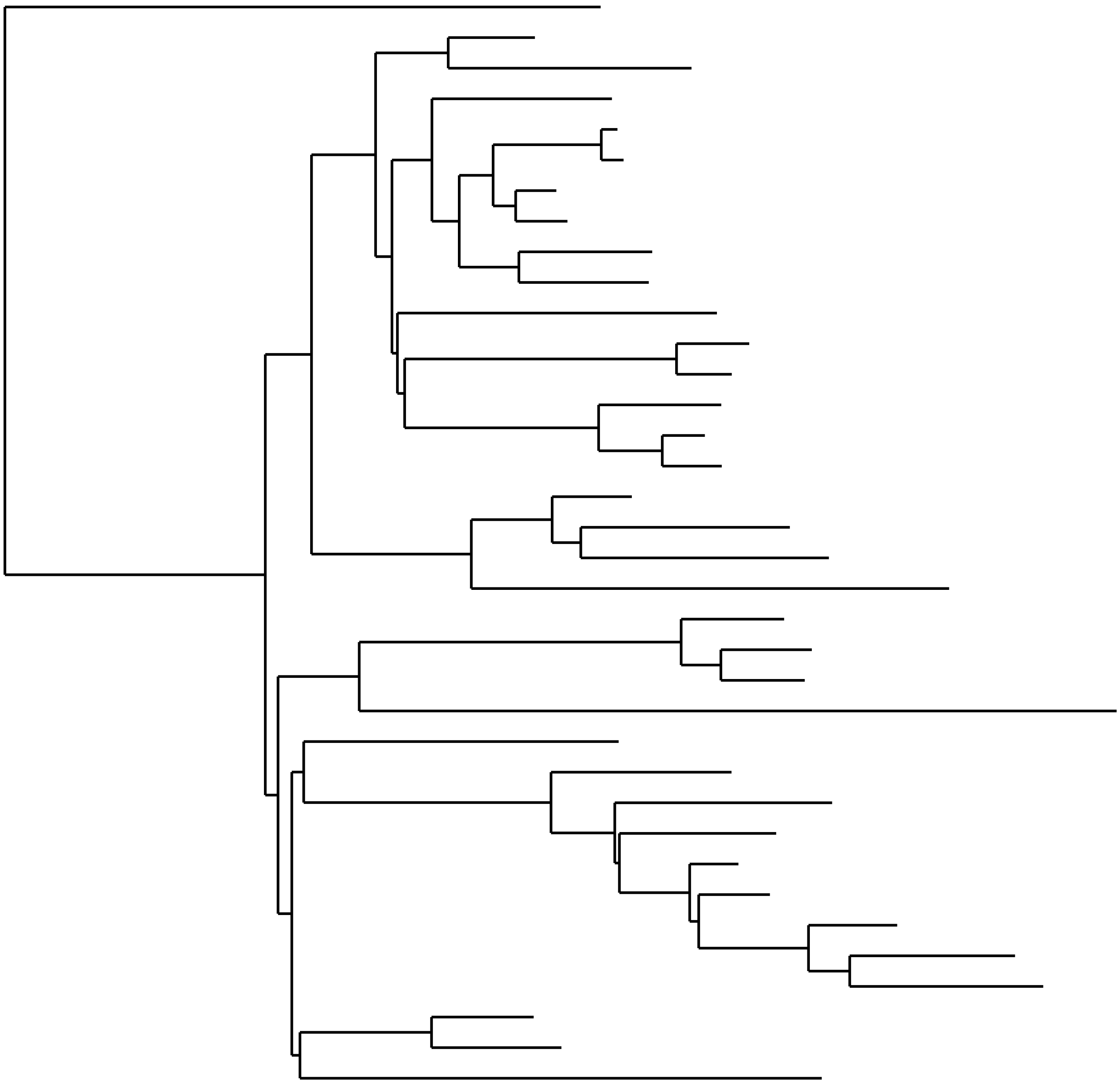

orig_upgma

Vangay filtered UPGMA

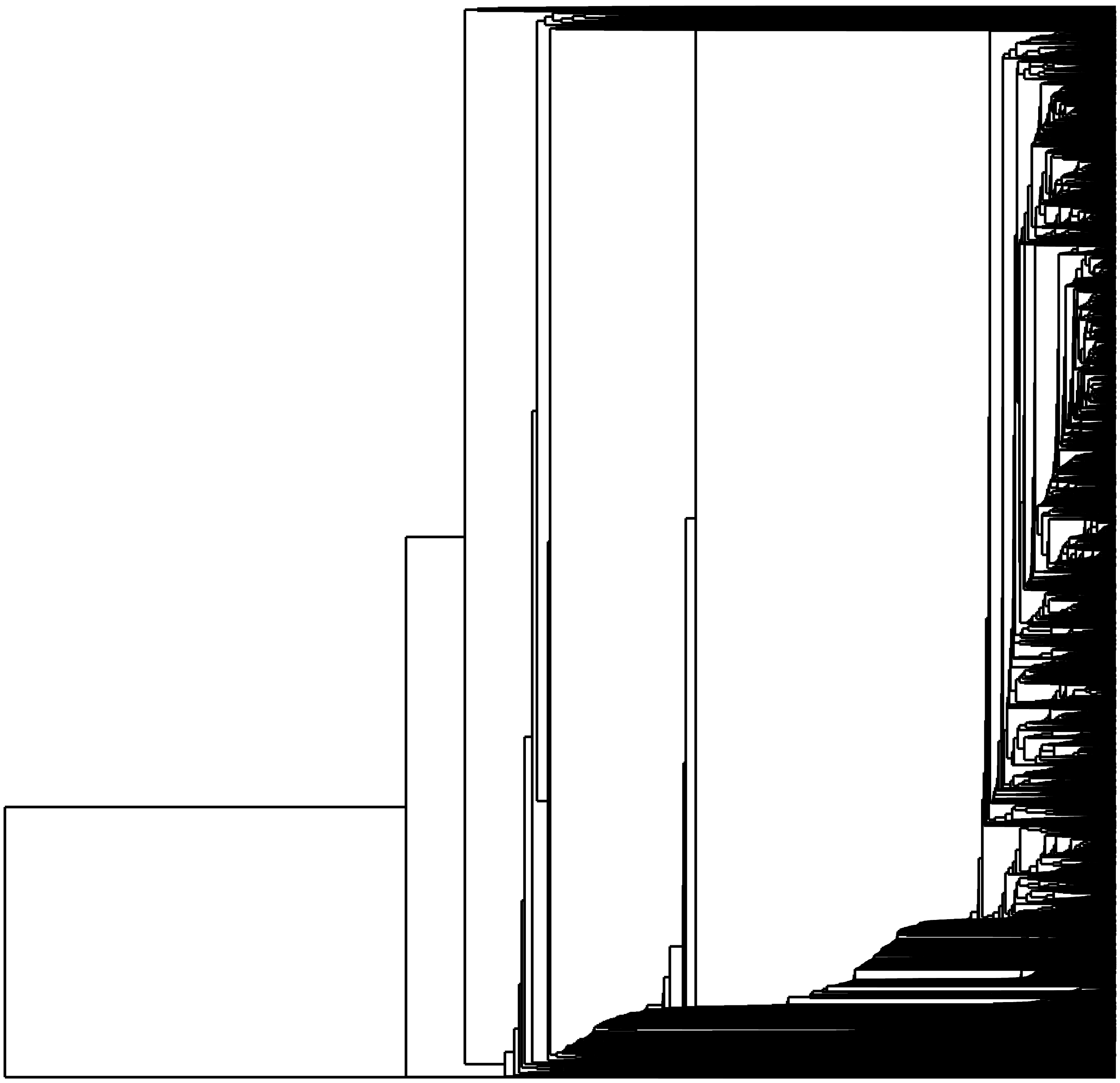

cln_upgma

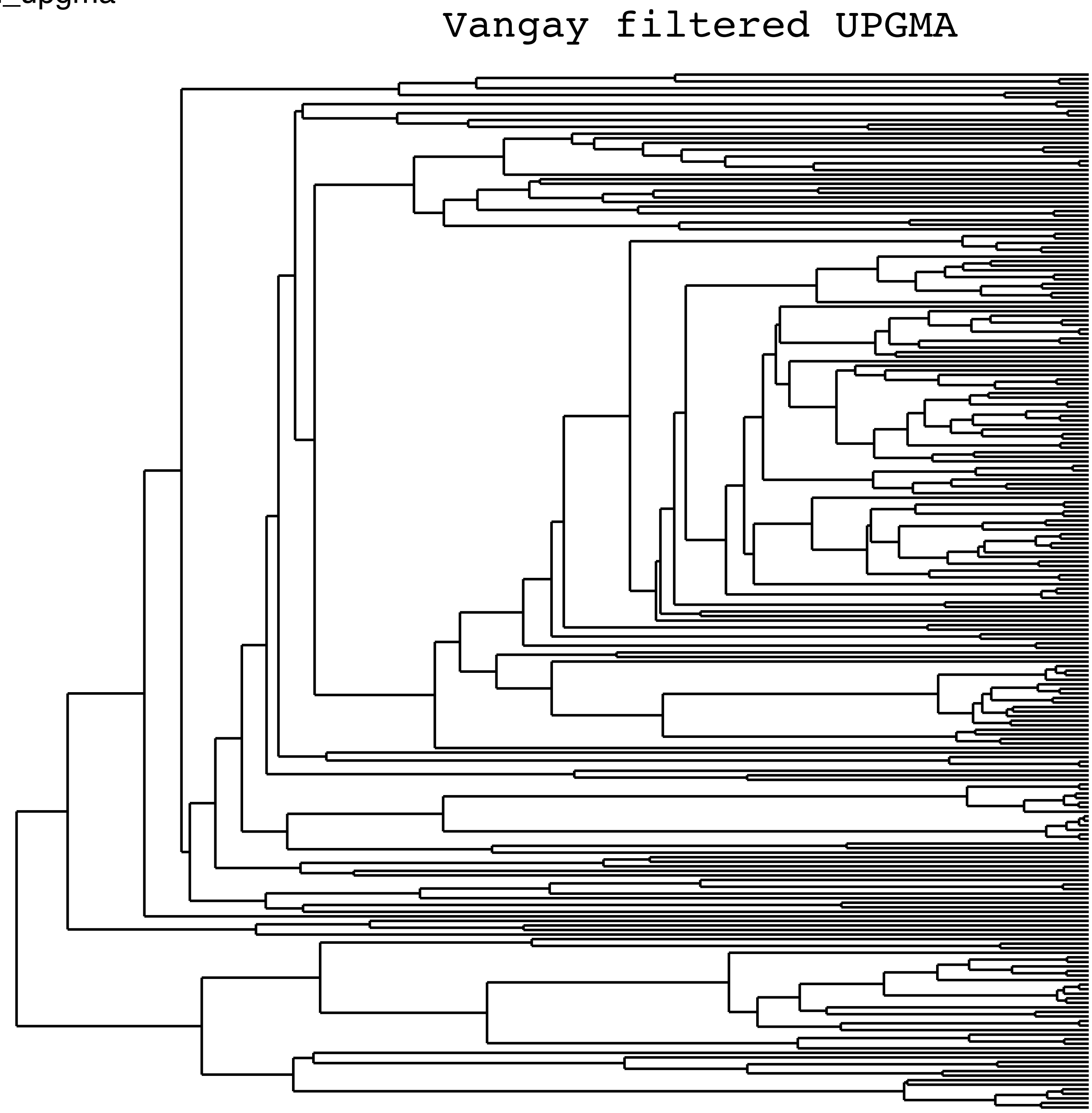

orig_iqtree

Vangay filtered IQTREE

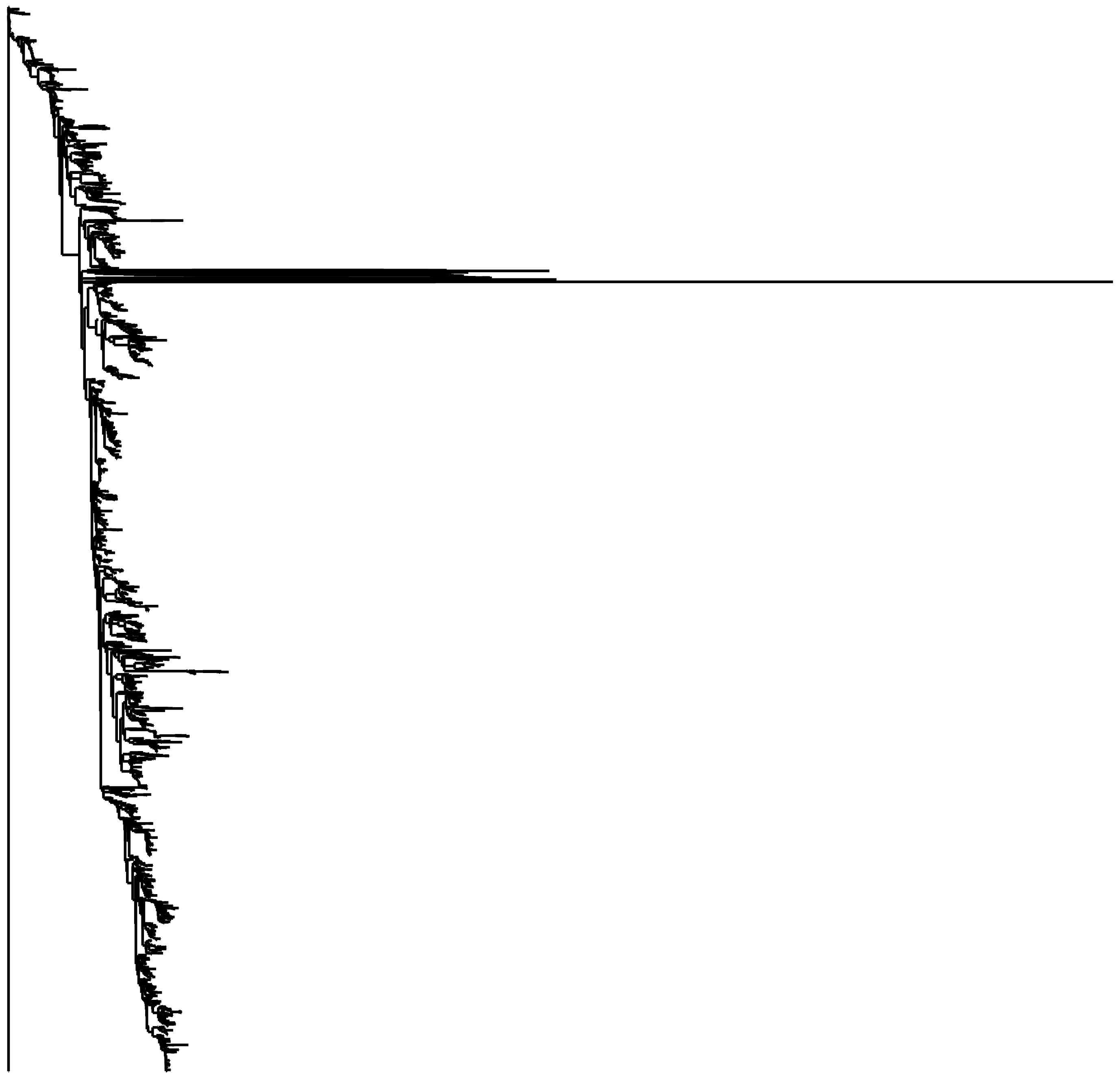

cln_iqtree

Vangay filtered IQTREE

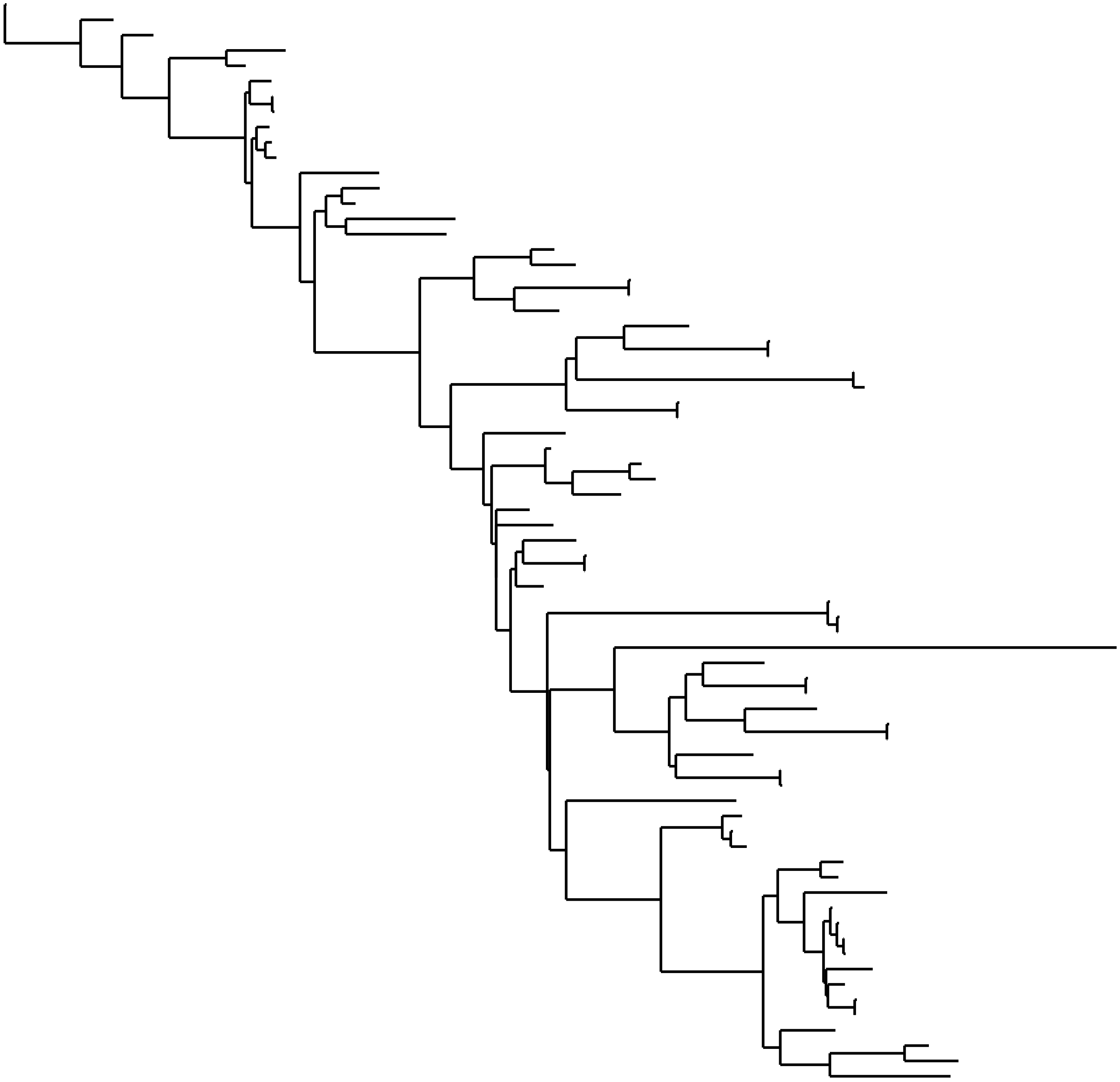

orig_ref

Noguera-Julian Silva LTP

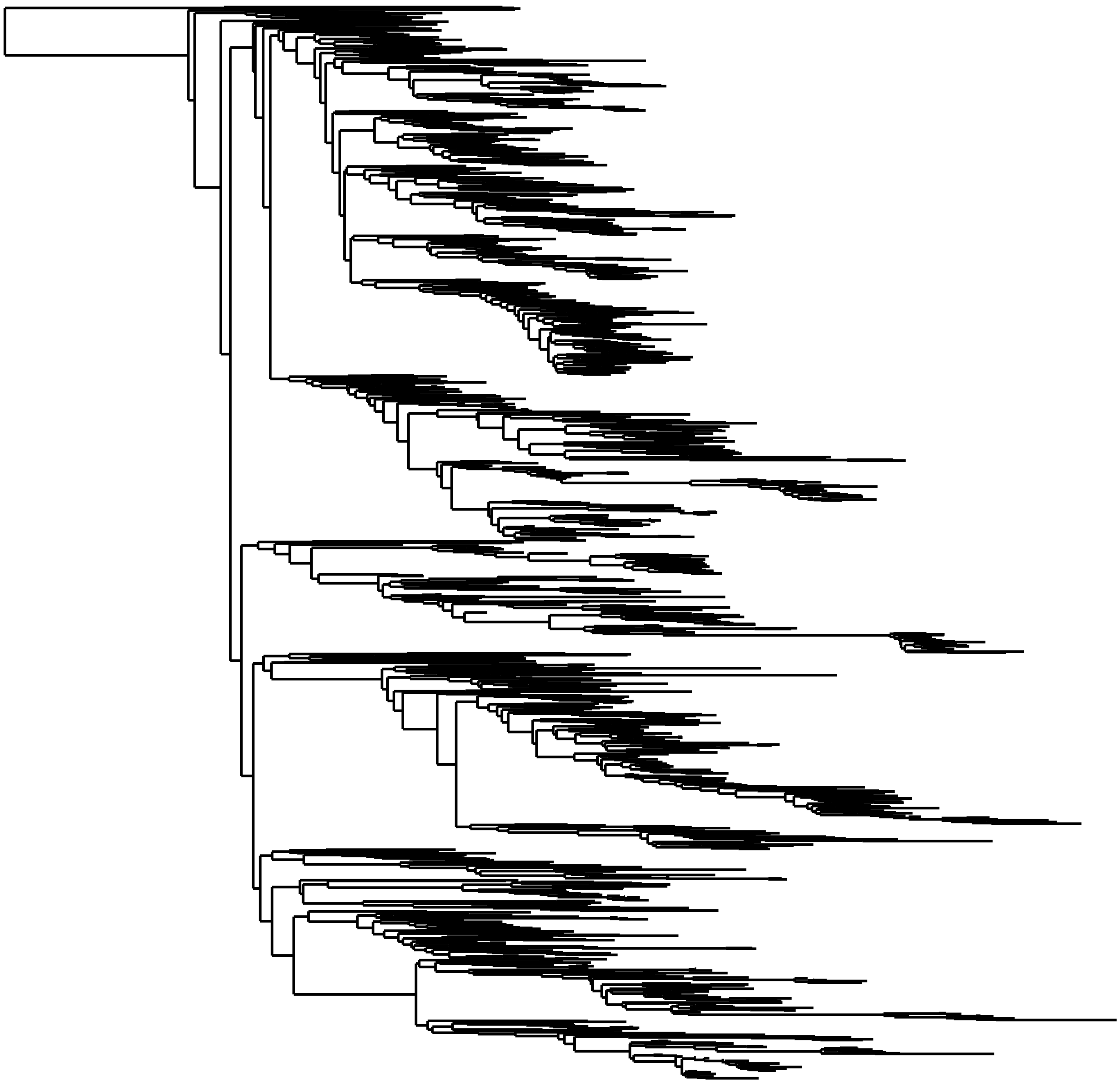

cln_ref

Noguera-Julian filtered Silva LTP

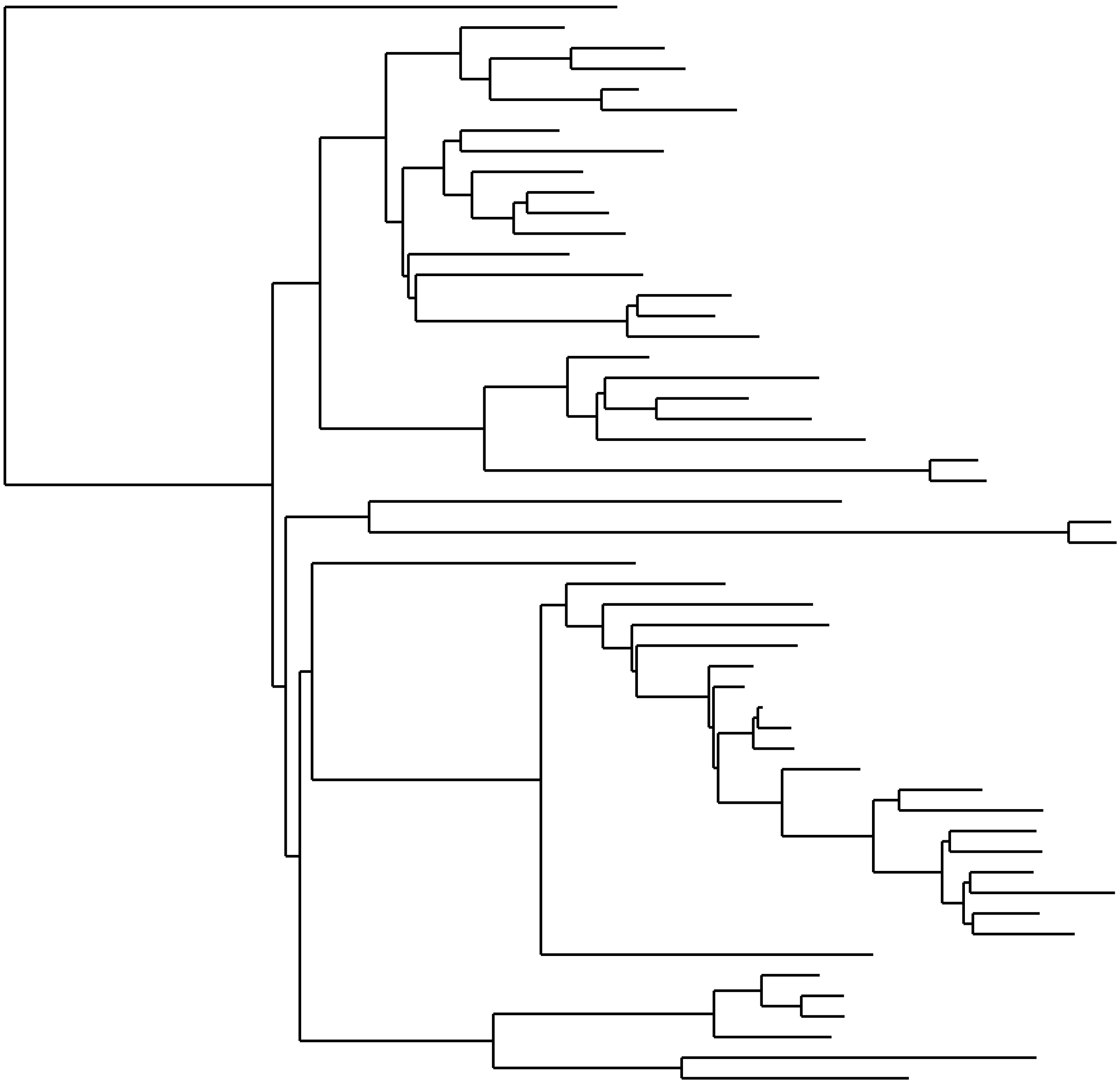

orig_upgma

Noguera-Julian UPGMA

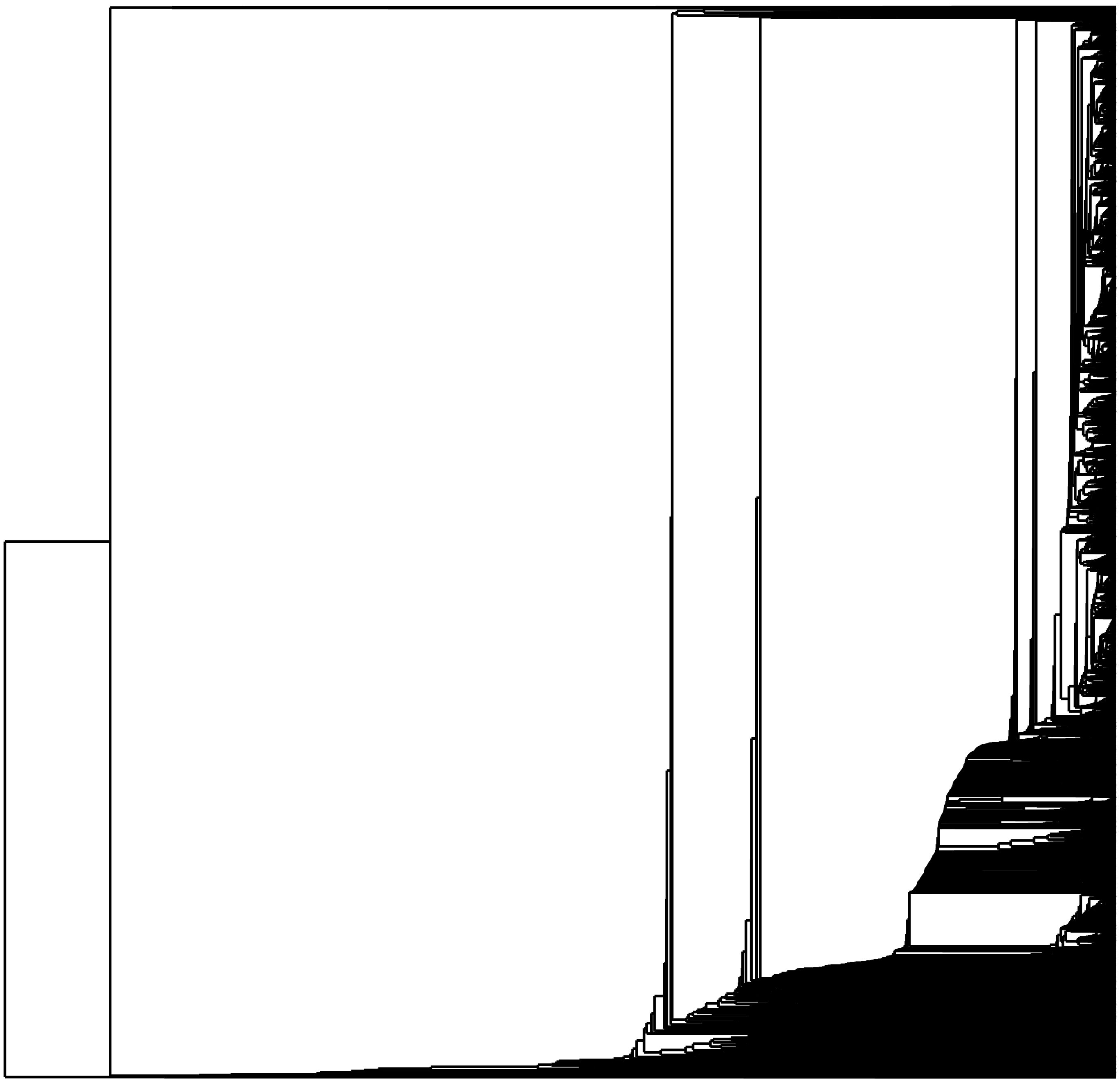

cln_upgma

Noguera-Julian filtered UPGMA

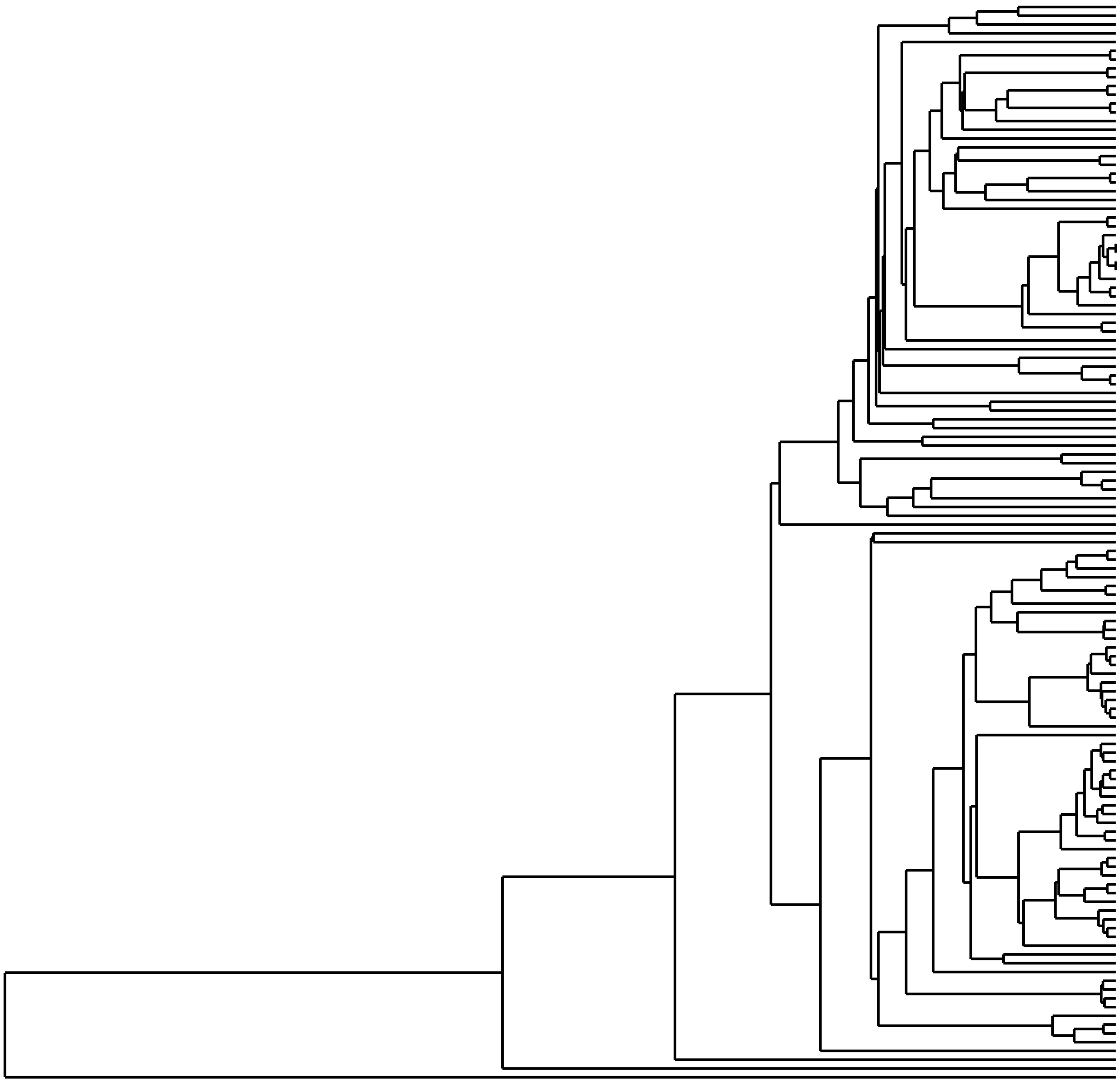

orig_iqtree

Noguera-Julian IQTREE

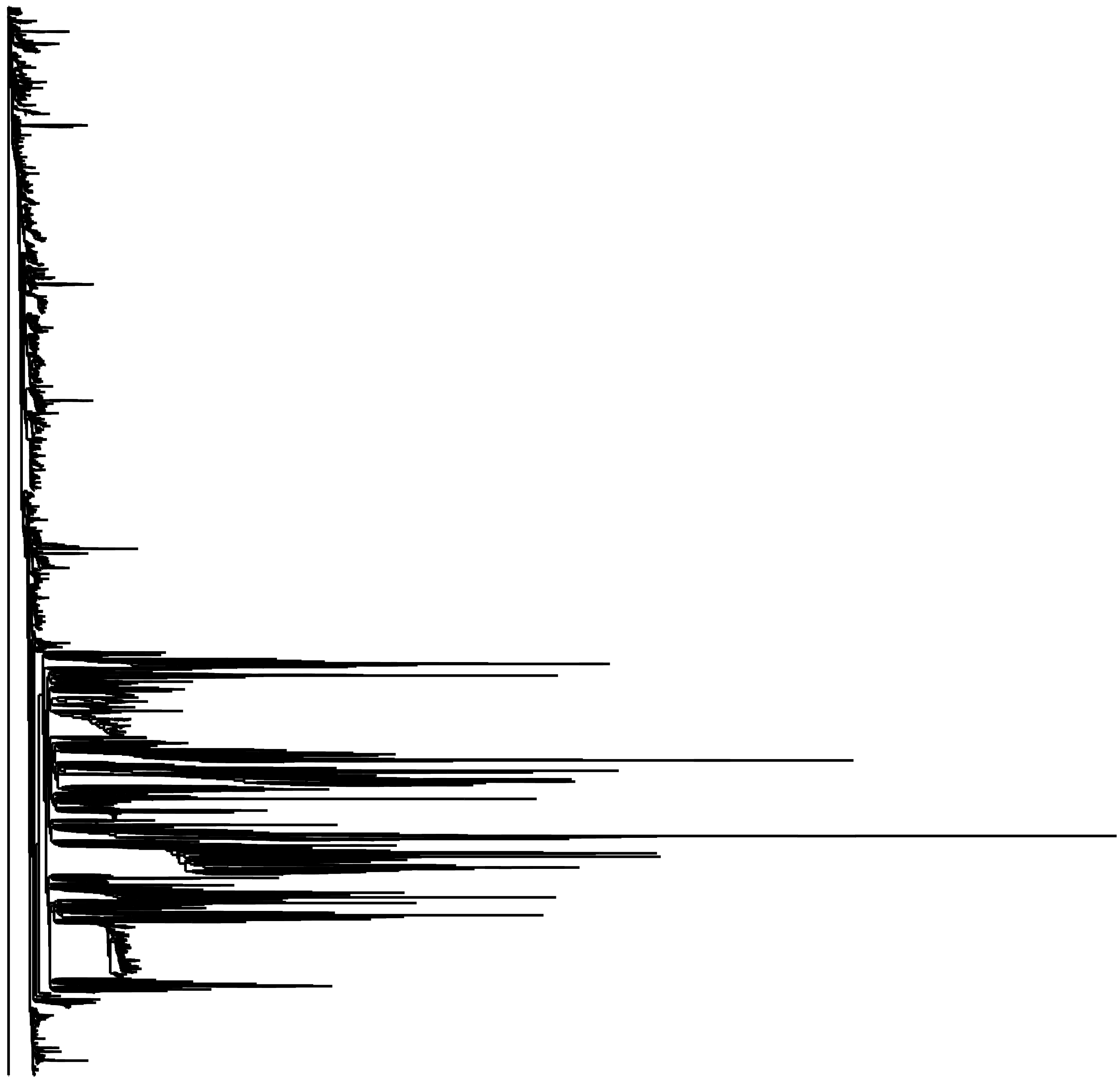

cln_iqtree

Noguera-Julian filtered IQT

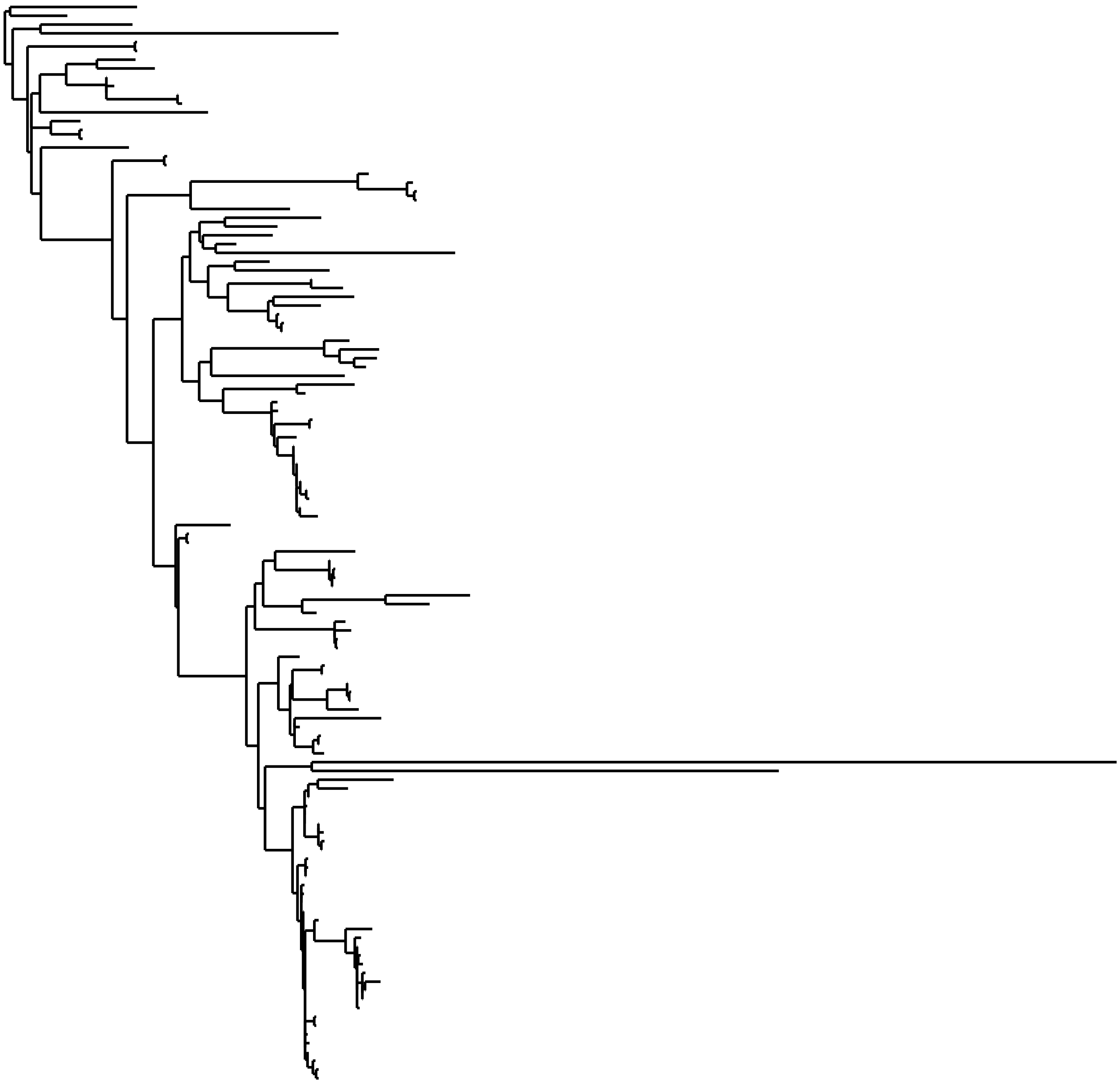

Supplement: Supplementary file 2 — Additional file 1: Supplementary A. Images of “true” trees. Images of the unfiltered SILVA LTP tree, the high variance/low abundance filtered SILVA LTP tree, the UPGMA, and IQTREE for each dataset (Vangay, Jones, Noguera-Julian, and Zeller). [file 40168_2023_1747_MOESM1_ESM.pdf]
