# Supplementary figures and images for: Proportion-based normalizations outperform compositional data transformations in machine learning applications
Source: Microbiome. 2024 Mar 5;12:45. doi: 10.1186/s40168-023-01747-z (PMC10913632; doi:10.1186/s40168-023-01747-z)

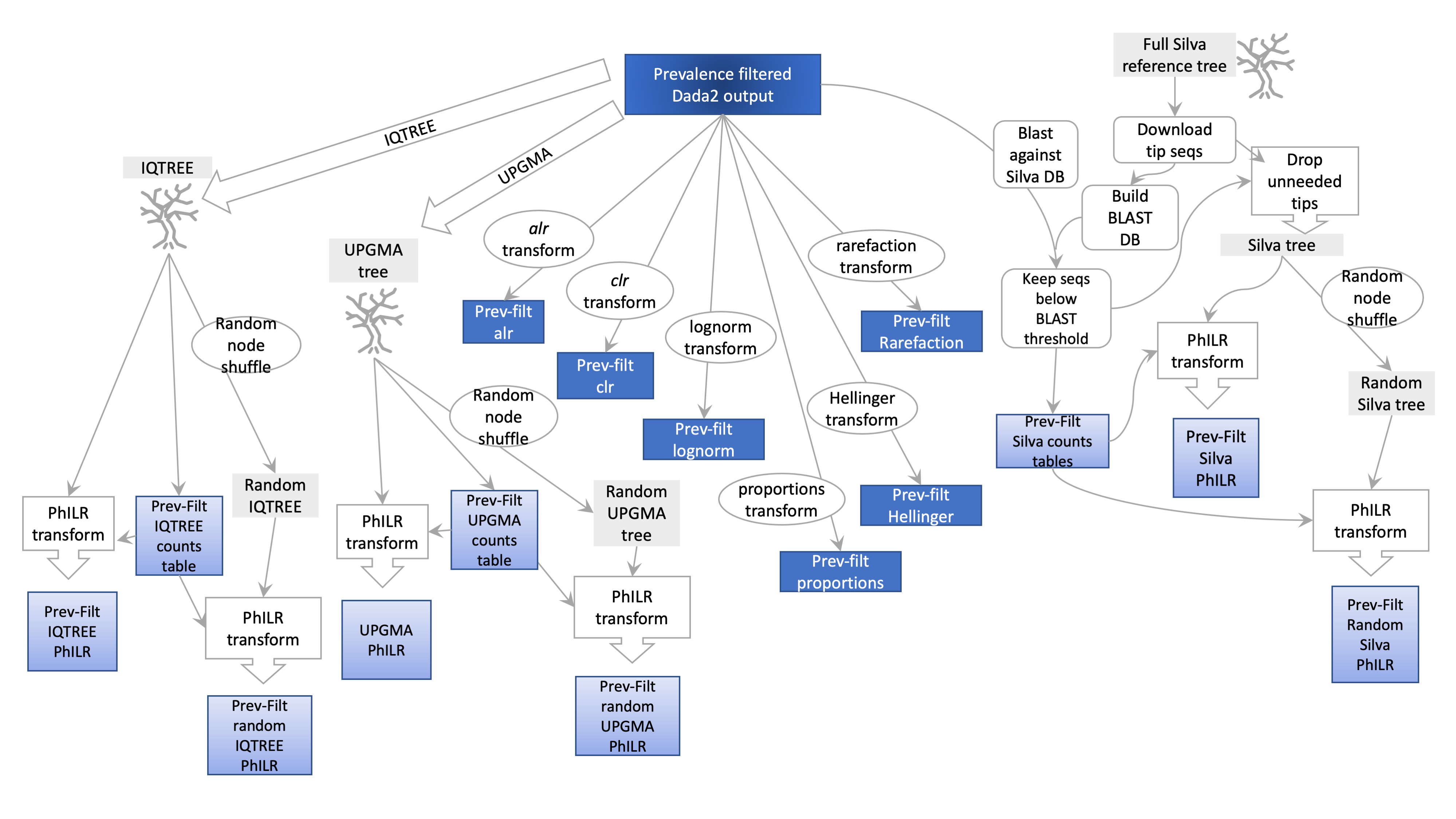

Supplement: Supplementary file 3 — Additional file 2: Supplementary B. Schematic representation of workflow for the creation of data transformations from the prevalence filtered Dada2 counts tables. This schematic starts with the prevalence filtered Dada2 counts tables and each step in the workflow (grey arrows with white bubbles) lead to trees (grey blocks) or final datasets (blue boxes) as described in the methods section. Datasets with random node shuffles were recreated at least 3 times. [file 40168_2023_1747_MOESM2_ESM.tiff]

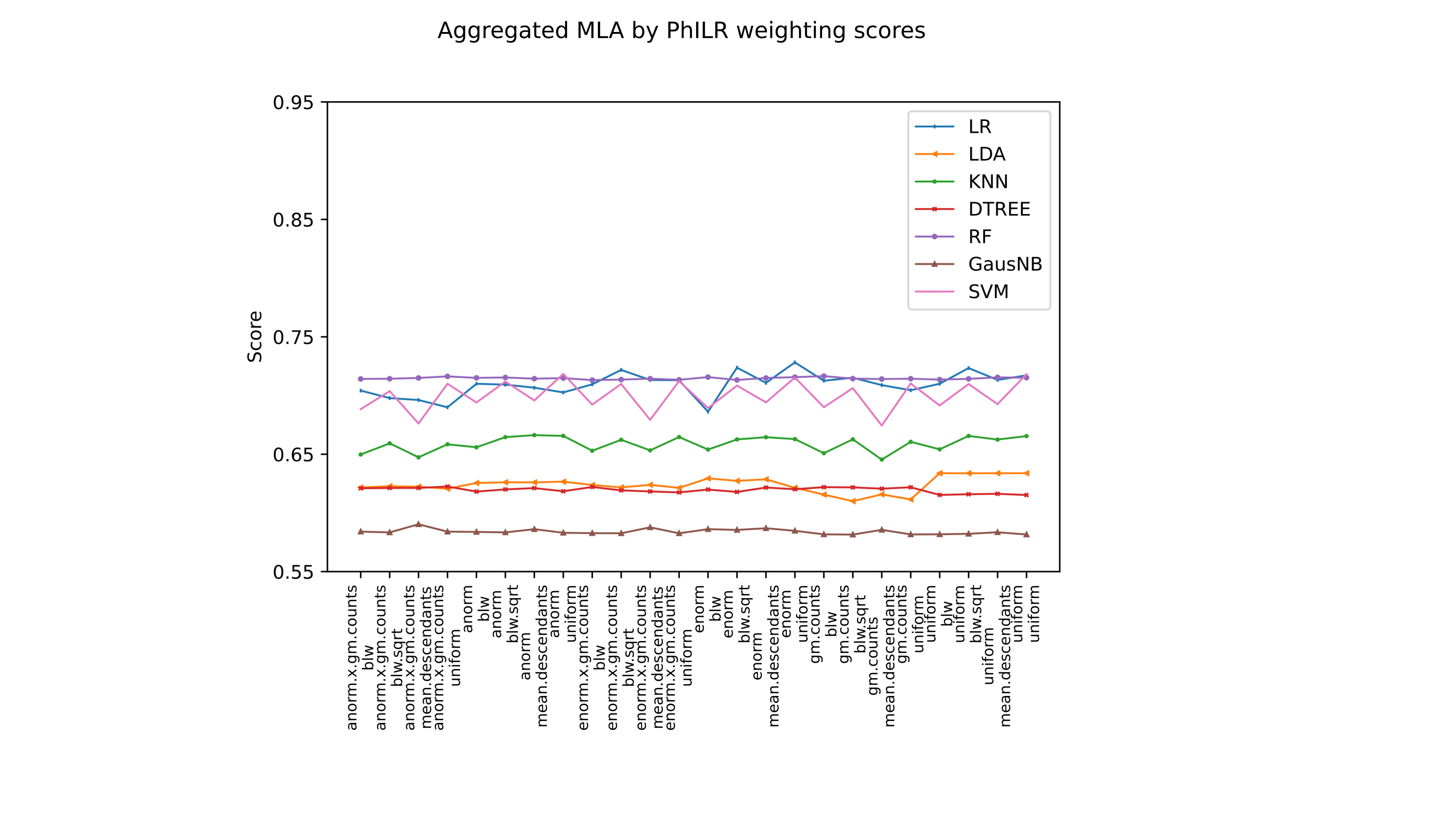

Supplement: Supplementary file 4 — Additional file 3: Supplementary C. Average scores of MLAs across PhILR weights from the Silva/LTP PhILR made from the raw counts table. To help determine which MLA to use for this project, we used 10-fold cross-validation to train and test each MLA on each PhILR weighting combination of for each of the 65 features. Within each dataset, for each PhILR combination, the means of all the features from each MLA (y-axis) were plotted showing the performance of each metadata feature for each PhILR weighting combination (x-axis). [file 40168_2023_1747_MOESM3_ESM.tiff]

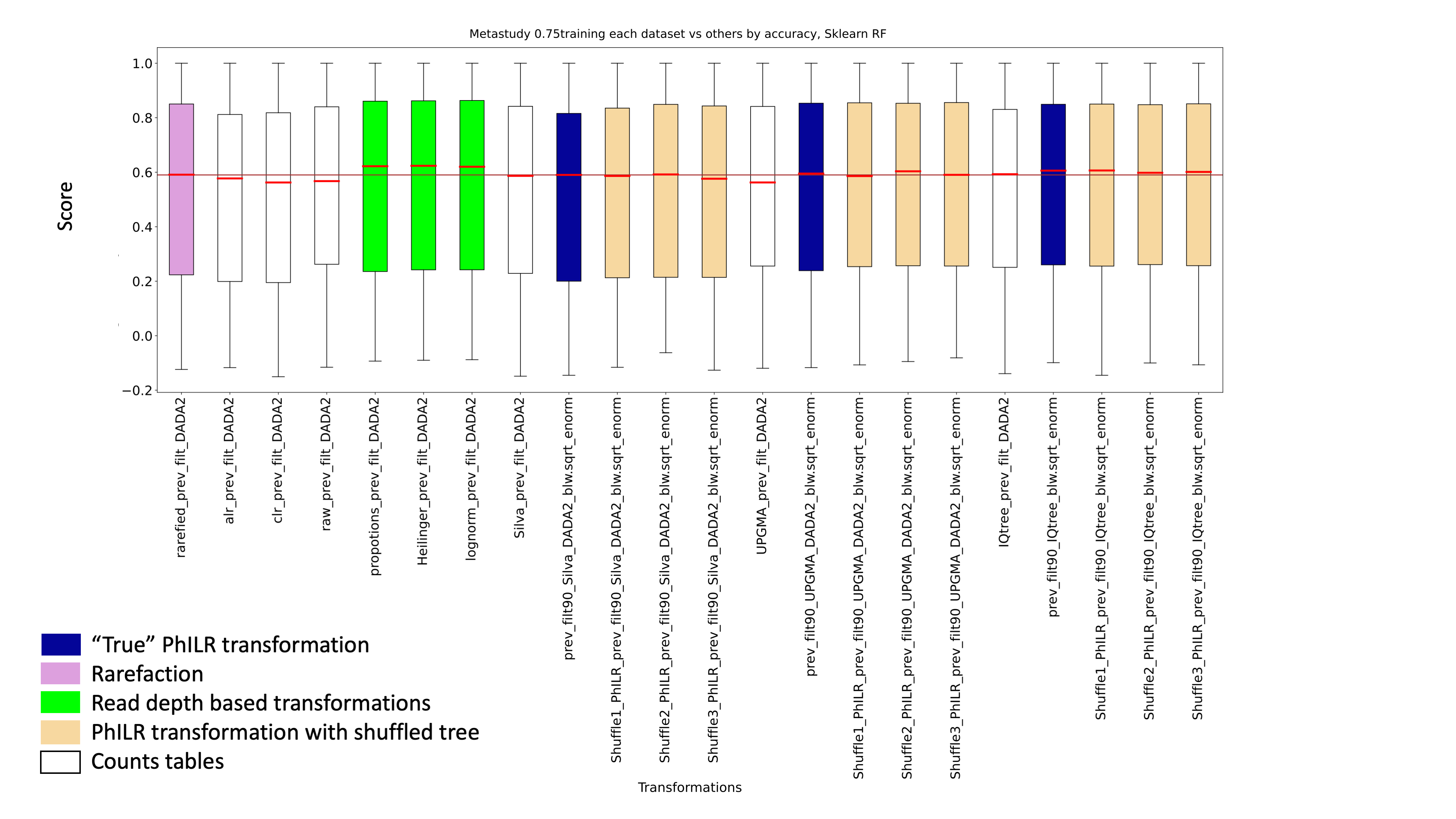

Supplement: Supplementary file 5 — Additional file 4: Supplementary D. For prevalence filtered tables, proportion-based transformations have the highest median accuracy. These box and whisker plots show the average of all the points in each metadata feature for each transformation (65 points for each transformation). The red bars represent the median for each transformation and the brown line represents the median of the entire dataset platform. [file 40168_2023_1747_MOESM4_ESM.tiff]
